# Dietary *Chlorella vulgaris* supplementation modulates health, microbiota and the response to oxidative stress of Atlantic salmon

**DOI:** 10.1038/s41598-024-72531-8

**Published:** 2024-10-10

**Authors:** Jonas Mueller, Doret R. van Muilekom, Jannick Ehlers, Marvin Suhr, Stéphanie C. Hornburg, Corinna Bang, Marie Wilkes, Thekla Schultheiß, Edmund Maser, Alexander Rebl, Tom Goldammer, Henrike Seibel, Carsten Schulz

**Affiliations:** 1 Department for Marine Aquaculture, Institute of Animal Breeding and Husbandry, Kiel University, Kiel, Germany; 2 Fraunhofer Research Institution for Individualized and Cell-Based Medical Engineering IMTE, Aquaculture and Aquatic Resources, Büsum, Germany; 3 Working Group Fish Genetics, Research Institute for Farm Animal Biology (FBN), Dummerstorf, Germany; 4 Institute of Animal Nutrition and Physiology, Kiel University, Kiel, Germany; 5 Institute of Clinical Molecular Biology, Kiel University, Kiel, Germany; 6 Institute of Toxicology and Pharmacology for Natural Scientists, University Medical School Schleswig-Holstein, Kiel, Germany; 7 Faculty of Agriculture and Environmental Sciences, University of Rostock, Rostock, Germany

**Keywords:** Atlantic salmon, Chlorella, Microalgae, Functional feed, Microbiome, Immunity, Oxidative stress, Immunology, Microbiology, Molecular biology, Physiology

## Abstract

Microalgae are emerging as functional feed ingredients in aquaculture due to their immune-stimulating and stress-modulating properties. We investigated the potential of the microalgae *Chlorella vulgaris* as a feed supplement to improve the health and modulate microbiota and stress responses of Atlantic salmon. Triplicate groups of Atlantic salmon (~ 126 g) were reared in a recirculating aquaculture system (RAS) at 15 °C and received diets supplemented with 2% (CV2) or 14% (CV14) spray-dried *C. vulgaris* daily, 14% once weekly (CV14w), or a control diet (CD) for 8 weeks. Subsequently, all groups were exposed to an acute one-hour peracetic acid (CH_3_CO_3_H; PAA) treatment, a commonly used disinfectant in RAS. While CV14 increased feed conversion (FCR) significantly, feeding the diets CV2 and CV14w improved protein retention efficiency. CV14 significantly modulated beta-diversity in the intestinal digesta and mucosa, but this effect was already visible in fish fed CV2. Feeding CV14 and, to a lesser degree, CV2 increased the relative abundances of *Paenarthrobacter* and *Trichococcus* in the digesta and mucosa, which are able to metabolize complex carbohydrates. However, the same diets reduced the abundance of the lactic acid bacteria *Lactobacillus* and *Weissella* in the digesta and *Floricoccus* in the mucosa. Peracetic acid exposure induced systemic stress (increase in plasma glucose and cortisol) and a local immune response in the gill, with the most prominent upregulation of several immune- and stress-regulated genes (*clra*, *cebpb*, *marco*, *tnfrsf14*, *ikba*, *c1ql2*, *drtp1*) 18 h after exposure in fish fed the control diet. Fish receiving CV14 once a week showed a reduced transcriptional response to PAA exposure. Catalase protein abundance in the liver increased following exposure to PAA, while superoxide dismutase abundance in the gill and liver was increased in response to *C. vulgaris* inclusion before stress. Overall, the results highlight that a high (14%) inclusion rate of *C. vulgaris* in feed for Atlantic salmon impairs feed conversion and shifts the intestinal microbiota composition in digesta and mucosa. Weekly feeding of *C. vulgaris* proves a viable approach in improving protein retention and improving transcriptional resilience towards oxidative stress in increasingly intensive production systems. Thereby this study may motivate future studies on optimizing temporal feeding schedules for health-promoting aquafeeds.

## Introduction

Aquaculture production continues to grow at a high rate, providing fish and seafood for a continuously increasing consumer demand globally^1^. A more sustainable intensification of fish farming is needed in order to ensure future growth of the industry. Implementing novel feed ingredients with less reliance on finite marine resources^2^, as well as adopting new farming practices with minimized environmental impacts^3^, are considered key to improve aquaculture sustainability. Furthermore, promoting fish health is a prerequisite for increasing production.

Atlantic salmon (*Salmo salar*) is one of the most valued fish species for human consumption, with a global production of 2.7 M tons in 2020^4^. In recent years, Atlantic salmon farming has been suffering from compromised health and high mortality in the sea phase of production^5,6^. This has led to a strong increase in the use of land-based recirculating aquaculture systems (RAS), in which the production environment can be fully controlled^7,8^. However, the RAS environment can pose challenges related to fish health, e.g. caused by high stocking densities^9^, reduced water quality^10^ as well as routine disinfection^11,12^. While the latter is used to maintain a high level of biosecurity within the system, disinfectants and their derivatives can induce a stress and an immune response in treated fish. Peracetic acid (CH_3_CO_3_H; PAA) is an oxidative biocide with broad anti-bacterial activity, which is commonly used due to its low effective dose, rapid degradation, and minimal environmental impact^12^. However, acute exposure to PAA was shown to induce the expression of antioxidant genes (*gpx, sod*) in the gill and increase plasma cortisol and antioxidant capacity of Atlantic salmon^13^. Furthermore, chronic PAA exposure induced the expression of immune genes in the olfactory rosettes in Atlantic salmon^14^ and increased serum antioxidant capacity and levels of free radicals in the gill and serum of rainbow trout^15^.

Functional feeds are reported to improve the overall health status of fish. Their beneficial stress-mitigating and immune-modulating properties have the potential to counteract negative side effects when fish are treated with PAA^16^. Microalgae have emerged as valuable functional feed ingredients because of their high content of functional compounds and nutritional value^17^. A variety of vitamins, pigments, polyphenols, and complex carbohydrates in microalgae contribute to their antioxidant, antimicrobial, and immune-modulatory activity^18–20^. *Chlorella vulgaris* is a widely used microalgae species in aquaculture due to its low production cost, high content of protein and functional compounds including lutein, ß-carotene, phytosterols, and vitamin E^21^. In our previous study, we demonstrated that diets enriched with 8% *Chlorella vulgaris* induced anti-inflammatory responses (reduction of *saa5* in the liver, *nfkbia* in the spleen, and *il10rb* and *il1r2* in the intestine) in Atlantic salmon^22^. Likewise, the activity of antioxidant enzymes (superoxide-dismutase, catalase, and glutathione peroxidase) and innate immune responses (lysozyme, respiratory burst activity and total antibody counts) were increased in plasma of Nile tilapia when fed with diets supplemented with 1.5% *Chlorella vulgaris*^23^. However, potential effects of microalgae on the immune system seem to depend on the inclusion level as shown in Nile tilapia^24^ and rainbow trout^25^, where high inclusion levels stimulated innate immunity. Optimal inclusion levels for many fish and microalgae species remain currently unknown.

Functional feeds not only directly affect the immune system of fish but also their intestinal microbiome, which is closely associated with animal health and performance^26,27^. Besides playing an important role in nutrient digestion, intestinal microbiota is involved in host immune responses as well as in the gut-brain axis^28–32^. The intestinal microbiota can be divided into transient (allochthonous) microbes associated with the digesta and resident (autochthonous) microbes residing within a complex matrix of mucin proteins^33^. The diet provides specific substrates for bacteria colonizing the digesta, shaping diet-dependent ecological niches. For example, plant-based diets have been associated with the occurrence of lactic acid bacteria of the phyla *Firmicutes* in the digesta of rainbow trout^34^ and Atlantic salmon^35^. Bacteria residing within the intestinal mucus are thought to be less influenced by diet^36^, but rather in intimate association with the host’s immune system. Whether functional diets enriched with *Chlorella vulgaris* can modulate both the microbiota of digesta and intestinal mucosa, as well as immunity of Atlantic salmon, remains currently unknown.

This study aimed to investigate the use of *Chlorella vulgaris* as a functional feed additive in diets for Atlantic salmon. More specifically, we hypothesized that the inclusion rate (2% vs. 14%) and mode of application (daily vs. weekly) of *C. vulgaris* fed to Atlantic salmon would influence growth performance and the microbiota of the digesta as well as intestinal mucosa. Furthermore, feeding the functional enriched diets was hypothesized to counteract stress- and immune-related effects caused by treatment with PAA as an oxidative stressor. A higher (14%) and lower inclusion rate (2%) was chosen in this study based on our previous findings^22^. This further allowed a comparison with an additional application of 14% once weekly, reflecting similar daily as well as weekly doses compared to the other treatments.

## Material and methods

### Feed and experimental groups

Three different diets were formulated (on dry matter basis) for Atlantic salmon^37^ containing different levels of the microalgae *Chlorella vulgaris*: a control diet (CD) without microalgae addition, a diet containing 2% *C. vulgaris* (CV2) and a diet containing 14% *C. vulgaris* (CV14). A fourth experimental group was established, which received 14% *C. vulgaris* only once per week (CV14w). The diets were designed to replace various plant-based components with the microalgae, as indicated in Table 1. The feed was cold-pressed into pellets of 4 mm diameter (Type 14U175, Amandus Kahl, Hamburg, Germany) at temperatures below 60 °C. This procedure was chosen over extrusion to minimize loss of functional compounds present in the microalgae. The produced feed was air-dried for 48 h, sieved and subsequently stored at 4 °C.

**Table 1:** Feed formulation and proximate composition of experimental feeds in g/100 g dry matter (DM).

Ingredients (g/100 g DM)	CD	CV2	CV14
Fish meal^1^	15	15	15
Blood meal^2^	6	6	6
Gelatine^3^	5	5	5
Pea protein isolate^4^	6	6	6
Soy protein concentrate^5^	18	18	10.65
Wheat gluten^6^	11.75	10.50	8.60
Wheat starch^6^	15	14.45	13
Canola oil^7^	7	7	7
Fish oil^1^	5	5	5
Palm oil	7	7	6.3
Biolysine^8^	0.6	0.6	0.5
Methionine^8^	0.15	0.15	0.15
Vitamin & mineral premix^4^	0.5	0.5	0.5
CaHPO_4_^9^	2	2	2
Cellulose^10^	1.0	0.8	0.3
Microalgae meal (*C. vulgaris*)^11^	0	2	14
Proximate comp. (g/100 g DM)			
Dry matter	92.3	88.3	89.4
Crude protein	49.8	49.9	50.1
Crude lipid	22.4	22.4	22.6
Crude ash	6.4	6.7	7.4
NfE^12^	21.4	21.1	19.9
Crude energy (MJ/kg)	24.2	24.2	24.2

^1^Fish meal & oil from mainly herring and sprat, Bioceval GmBH & Co. KG, Cuxhaven; Germany; ^2^Saria SE & Co. KG, Selm, Germany; ^3^Gustav Ehlert GmbH & Co. KG, Verl, Germany; ^4^Emsland-Aller Aqua GmbH, Golßen, Germany; ^5^EURODUNA Rohstoffe GmbH, Barmstedt, Germany, ^6^Kröner-Stärke GmbH, Ibbenbüren, Germany; ^7^Cargill GmbH, Riesa, Germany; ^8^Evonik Industries AG, Esssen, Germany, ^9^Lehmann & Voss & Co. KG, Hamburg, Germany ^10^Mikro-Technik GmbH & Co. KG, Bürgstadt am Main, Germany; ^11^Microganic GmbH, Melle, Germany; ^12^NfE (nitrogen-free extract) = 100–(crude protein + crude lipid + crude ash); DM (dry matter).

### Experimental setup

Atlantic salmon post-smolts were sourced from Danish Salmon A/S (Hirtshals, Denmark) and allowed to acclimate for one month in a recirculating aquaculture system (RAS). The RAS (24 m3, turnover rate 4 times h^−1^) was equipped with a drum filter (mesh size 40 μm, type KTS 8-12, Kunststoff Spranger, Plauen, Germany), a moving bed biofilter (Kunststoff Spranger), a protein skimmer (FLOTOR, Kunststoff Spranger) and an UV disinfection system (Aqua medic, Cologne, Germany) for water treatment. Oxygen was supplied through an oxygen cone, with additional air supplied in the tanks. Water quality was maintained in a suitable range for Atlantic salmon over the trial period (20.9 ± 2.1 psu, 15.1 ± 0.4 °C, 7.4 ± 0.2 pH, 10.6 ± 0.2 mg/L O_2_, 0.07 ± 0.09 mg N/L NH_4_^+^, 0.05 ± 0.02 mg N/L NO_2_^-^). During the time of acclimation, the fish were fed twice daily by hand, at ~ 1% of their bodyweight, a commercial salmon diet (Aller Aqua A/S, Christiansfeld, Denmark). Prior to the start of the experiment, Atlantic salmon (~ 126 g) were randomly allocated in groups of 20 individuals into 12 tanks (150 L) of the RAS. Triplicate tanks were randomly assigned to receive either the control diet (CD), the diet with 2% (CV2) or 14% (CV14) *C. vulgaris* on a daily basis, or 14% *C. vulgaris* once weekly (CV14w) while receiving the CD diet during the other days. Weekly feeding of CV14 occurred on day 4 of each experimental week during the trial. All fish were fed twice daily (9 a.m. and 2 p.m.) by hand for 56 feeding days at 1% of their body weight. The ration size was chosen to ensure all groups consume the delivered feed and effects due to differences in voluntary feed intake among groups can be excluded. Daily feed rations were adapted based on growth performance acquired after group weighing in weeks two and five of the experiment, during which two days the fish were not fed.

### PAA treatment and sampling

Samples were collected before the start (T0) and after eight weeks of feeding the experimental diets (T1). Following this sampling, all groups were subjected to acute oxidative stress induced by treatment with peracetic acid (PAA; WOFA-steril classic, Kesla, Germany) at 2.5 µL/L water, which is commonly applied for prophylactic treatment in RAS^12,38^. The product is a stabilized PAA solution with 40% PAA and 12% H_2_O_2_. For this purpose, the water inlet of the tank was closed, while continuous aeration in the tank ensured oxygen levels were maintained above 85% saturation. Then 375 µL of PAA solution was added to the tank, and the water inflow was opened again after 1 h. All groups were subsequently sampled 1 h after stress (T2) and 18 h after stress (T3).

For all three samplings, 12 fish per treatment (4 per tank) were netted from the experimental tanks and euthanized by buffered MS-222 (0.3 mg/L). Total length and weight were recorded for each fish and a 2 mL blood sample was collected from the caudal vein using heparinized syringes. The blood was centrifuged at 4000 g and 4 °C for 8 min and aliquots of the plasma were frozen on dry ice and stored at − 80 °C.

During the samplings T1 and T3, the fish were laterally, opened and the organs were carefully removed. The liver and spleen were weighed for the calculation of organ-specific indices. A piece of the liver, as well as the second left gill arch, were removed and frozen on dry ice for later protein extraction. The head kidney and first left gill arch were removed, placed in a RNAse-free tube and frozen in liquid nitrogen for subsequent gene expression analysis.

At T0 and T1, the intestine was removed from the abdominal cavity using sterile tools and digesta of the distal intestine were squeezed into 2 mL sterile collection tubes and frozen on dry ice. Remaining digesta were removed by flushing the intestine with 5 mL of sterile PBS. Subsequently the intestine was longitudinally opened and mucus of the distal intestine was collected by scraping over the intestinal surface with a sterile spatula. Intestinal mucus samples, hereafter referred to as mucosa, were frozen and all samples were stored at − 80 °C until DNA extraction.

At both sampling points T0 and T1 four fish per tank were pooled for the analysis of whole-body proximate composition.

### Calculations

To evaluate the effects of the different diets on the growth performance and organ indices of the fish, several indices were calculated as follows: SGR (specific growth rate) = (ln (weight_final_) – ln (weight_initial_)) / feeding days × 100; TGC (Thermal growth coefficient) = (weight_final_^1/3^–weight_initial_^1/3^) × 1000 / sum (water temperatures per feeding day); FCR (feed conversion ratio) = total feed intake (g) / weight gain (g); PER (protein efficiency ratio) = weight gain (g) / crude protein intake (g); PRE (protein retention efficiency) = crude protein gained (g) / crude protein intake (g) × 100; CF (Fulton´s condition factor) = fish weight / fish length^3^ × 100; HSI (hepatosomatic index) = liver weight (g) / fish weight (g) × 100; SSI (spleen somatic index) = spleen weight (g) / fish weight (g) × 100.

### Proximate composition of whole body and diets

The proximate composition was assessed in diets (Table 1) and whole-body homogenates in duplicate using identical procedures. The whole-body samples underwent freeze-drying (Alpha 1–2 LD plus and Alpha 1–4 LSC, Martin Christ Gefriertrocknungsanlagen GmbH, Osterode am Harz, Germany) until a consistent weight was achieved. Subsequently, the samples were homogenized with a knife mill (GM 200, Retsch GmbH, Haan, Germany). The analysis of nutrients and gross energy was performed following EU guideline (EC) 152/2009. The determination of dry matter content involved drying the samples at 103 °C in a drying oven for 4 h (ED 53, Binder GmbH, Tuttlingen, Germany). Ash content was established by subjecting the samples to combustion at 550 °C (P300, Nabertherm, Lilienthal, Germany). The Kjeldahl method was used to assess crude protein content (InKjel 1225 M, WD30, Behr, Düsseldorf, Germany). Crude lipids were extracted using petroleum ether within a Soxhlet extraction system (Soxtherm, Hydrotherm, Gerhardt Königswinter, Germany) and crude lipid content was subsequently quantified gravimetrically. Lastly, gross energy was quantified using a bomb calorimeter (C 200, IKA, Staufen, Germany).

### Plasma stress and health indicators

Plasma ions (Na^+^, Cl^-^) were determined potentiometrically using commercial dry chem slides on a Fuji Dry Chem NX500i (Fujifilm, Tokyo, Japan). 50 µl plasma from each individual was used for the plasma ion analysis and 10 µl for the remaining parameters. Plasma glucose was determined on a Fuji Dry Chem NX500i (Fujifilm) using kits from the manufacturer. Free cortisol in plasma samples was measured using a commercial Enzyme-linked Immunosorbent Assay (ELISA) Kit (Demeditec Diagnostics GmbH, Kiel, Germany).

### Protein quantification in liver and gill

Protein concentrations of catalase (Cat) and Cu, Zn superoxide dismutase (Sod1) were analysed in salmon liver and gills. The protein concentrations in the tissue samples were examined with SDS-PAGE and Western Blot. The cytoskeletal filament protein ß-actin served as the housekeeping protein for the loading control. A no template control (nuclease-free water) as well as a positive control per antibody (*Danio rerio* liver for Cat, bovine liver for Sod1 and human HEK-293 cells for ß-actin) were run (Fig. S1). Tissue samples of four fish per tank were pooled for each organ and the total protein was extracted using radioimmunoprecipitation (RIPA) lysis buffer (RIPA Lysis Buffer System, Santa Cruz Biotechnology Inc., Dallas, TX, USA), according to the manufacturer’s protocol. A concentration of 20 μg extracted total protein per pooled sample, adjusted to 13 µL with nuclease-free water, was incubated in reducing conditions with 5 µL 4 × LDS sample buffer (mPAGE, EMD Millipore Corporation, Burlington, MA, USA) and 2 µL 1 M 1,4-Dithiothreit at 70 °C for 10 min. The SDS-PAGE was performed in an Xcell SureLock Mini-Cell (Thermo Fisher Scientific, Waltham, MA, USA) using precast 4–12% Bis–Tris gradient gels and MOPS SDS running buffer (both mPAGE, EMD Millipore Corporation, Burlington, MA, USA). The electrophoresis was performed at 140 V for 90 min. Afterwards, proteins were electro-transferred to a PVDF membrane in transfer buffer (mPAGE, EMD Millipore Corporation, Burlington, MA, USA) at 30 V for 100 min. For parallel protein detection of Cat and Sod1 the membrane was dried at room temperature, horizontally cut just below the 35 kDa marker, reactivated with 100% methanol, washed and blocked for 90 min at room temperature with 150 rpm in PBS-T containing 5% skim milk powder. Primary antibody incubation, with Cat antibody (ab209721, Abcam, Cambridge, UK) and Sod1 antibody (NBP2-24915, Novus Biologicals, Bio-Techne Ltd., Abingdon, UK) both in 1:500 dilution in PBS-T containing 2.5% skim milk, was performed at 4 °C at 150 rpm overnight. Secondary antibody anti-rabbit IgG conjugated HRP (sc-2357, Santa Cruz Biotechnology Inc, Dallas, TX, USA) was incubated in a 1:5000 dilution at 20 °C for 90 min at 150 rpm. Detection was performed using ECL detection reagents (Amersham, Global Life Sciences Solutions Operations UK Ltd, Little Chalfont, UK) and chemiluminescence film (Amersham, GE Healthcare Ltd, UK) with exposure times of 5 min for Cat and 20 s for Sod1. For the subsequent detection of ß-actin, the antibodies were stripped off the membrane using 100 mM Glycin buffer (pH 2.5) for 2 × 30 min at 20 °C with 150 rpm shaking. Afterwards, the membrane was incubated in a 1:5000 dilution of ß-actin antibody (NB600-503, Novus Biologicals, Bio-Techne Ltd.) at 4 °C with 150 rpm overnight. Quantification of protein abundance was done by densitometric analysis of the protein bands using GIMP and normalized to housekeeping protein abundance.

### Microbiota in digesta and mucosa: DNA extraction, sequencing and bioinformatic analysis

To evaluate the digesta and mucosa-associated bacterial communities of the intestine of individual fish, DNA from the digesta and mucosa samples was extracted using approximately 200 mg of digesta/mucosa sample as input for the QIAmp Fast DNA Stool Mini Kit (Qiagen, Germany, Cat. No. 51604) and QIAmp DNA Microbiome (Qiagen, Germany, Cat. No. 51704), respectively. Digesta material was transferred to tubes prefilled with 0.70 mm garnet beads and with 1 mL InhibitEx lysis buffer and bead beating was performed 2 × 45 s using a bead mill homogenizer (Fisherbrand™, Thermo Fisher Scientific, Waltham, Massachusetts, US). In addition, samples were incubated at 95 °C for 5 min and subsequently centrifuged for 3 min at 20,000 rcf. The following DNA extraction using the supernatant was conducted according to the manufacturer’s instructions. Mucosa material was extracted according to manufacturer’s instructions with no manual pre-processing of the samples. To exclude and verify potential contamination during DNA extraction, preparation blanks were included in the extraction process. For sequencing, all DNA samples were amplified by PCR targeting the 16S rRNA V3–V4 (341 F ‘CCTACGGGAGGCAGCAG’ and 805R ‘GGACTACHVGGGTWTCTAAT’^39^. Multiplexed samples were finally loaded on an Illumina MiSeq v3 platform 2 × 300 bp. A detailed description of PCR cycling conditions and sequencing has been published previously^40,41^.

The 16S rRNA gene sequences were analysed using the Linux-based version of Quantitative Insights Into Microbial Ecology 2 (Qiime2, v.2021.2.0; Bolyen et al.^42^). Leftover primers and spacers were removed from sequencing reads using Cutadapt and filtering for low-quality reads and chimeras as well as merging of paired-end reads was conducted using DADA2^43^ with truncation parameters of 277 and 221 for forward and reverse reads, respectively, and truncation quality cut-off of 2. ASVs with a frequency of  > 25 and an occurrence in at least two samples were retained for taxonomic determination using the SILVA reference database version 138.1^44^. Based on the taxonomic information, only ASVs that are of bacterial origin and have been identified at least up to the phylum level were kept for downstream analysis. In addition, ASVs from *Cyanobacteria* and mitochondrial origin were excluded from the dataset. To investigate the metabolic activity, Phylogenetic Investigation of Communities by Reconstruction of Unobserved States (PICRUSt2) was utilized to assess the functional profiles of the gut microbial community in Atlantic salmon, focusing on the composition of the 16S rRNA gene^45^. Metabolic potential of bacterial communities was inferred from the MetaCyc database.

### Gene expression in the head kidney and gill

After extracting the total RNA with TRIzol (Thermo Fisher Scientific), the ISOLATE II RNA Micro Kit (Meridian Bioscience Inc., Cincinnati, Ohio, USA) was used for its purification. The concentration and quality of isolated RNA was measured using NanoDrop One (Thermo Fisher Scientific). Reverse transcription of high-quality RNA into cDNA was then performed with the Reverse Transcription Master Mix (Standard BioTools, South San Francisco, California, USA). Fluidigm PreAmp Master Mix was used to preamplify cDNA samples, followed by a treatment with exonuclease I (New England BioLabs, Frankfurt/Main, Germany). The manufacturer's instructions were followed for all procedures.

Salmon’s immunocompetence was evaluated by profiling the expression of genes involved in key immunological and stress-related pathways^46^. The selected set of genes and their corresponding primers, accession codes and further details are described in Table S1. Multiplex quantitative Real-Time PCR (qPCR) was carried out on 48.48 Gene Expression biochips, which were initially primed in the MX IFC Controller (Standard BioTools). The inlets for samples and assays were filled with pre-amplified cDNA samples and primers. Then, following the manufacturer’s thermal protocol “GE Fast 48 × 48 PCR + Melt v2.pcl” (application type: gene expression; passive reference: ROX; assay: single probe), Biomark HD was used to determine the concentrations of selected transcripts. Fluidigm real-time PCR analysis software v3.0.2 (Standard BioTools) was used to obtain the raw Cq data and with this, the relative expression of the target genes was calculated by the ΔCt method. To normalize the results, three reference genes encoding β-actin (*actb*), ribosomal protein L4 (*rpl4*) and ribosomal protein S20 (*rps20*) were utilized. The mean Cq for all samples of every gene per organ served as a calibrator during the calculations. The fold changes were calculated per gene by dividing the relative expression after PAA treatment of a diet group by the relative expression before PAA treatment of the same diet group. The log2-transformed values of the calculated fold changes were used to construct the corresponding heatmaps.

### Statistical analysis

Statistical analysis and data visualization were performed in R (version 4.3.1; R Core Team 2021, Vienna, Austria) within the environment R studio. The significance threshold was set at α = 0.05. Statistical evaluation started with the definition of an appropriate statistical model followed by checking model assumptions employing graphical residual analysis. Upon this a Pseudo R^2^ was calculated^47^ and analysis of variance was conducted (ANOVA). Models based on generalized least squares (gls)^48^ were used to evaluate the effect of the diet on the production parameters (e.g. growth performance) and protein abundance in the gill and liver, which did not include a random (tank) effect, while for organ indices a linear mixed effect model (lme; Ref.^49^) including a random tank effect were used. Models for protein abundance (per organ) included the factor diet and timepoint and residuals were assumed to be normally distributed and heteroscedastic, while models for growth parameters included only the factor diet. Multiple contrast tests were conducted to compare among groups based on Tukey multiple comparisons accounting for p-value adjustment^50^. For alpha diversity parameters, Observed ASVs and Shannon diversity, within the respected sample type (digesta and intestinal mucus), a linear-mixed effect model with tank as a random factor was used for statistical evaluation. Residuals were assumed to be approximately normal distributed and heteroscedastic based on graphical residual analysis. For beta diversity, permutational multivariate analysis of variance (PERMANOVA) with Bray–Curtis distance as input was conducted using the *adonis2* function from the *vegan* package^51^ to test for statistical differences in the community composition according to the factor diet. Pairwise comparisons were conducted using a pairwise PERMANOVA. In addition, multivariate homogeneity of group dispersion was tested with factor diet using *betadisper* function from *vegan*. To infer key metabolic microbial pathways which were enriched in a specific diet PICRUSt2 analysis was performed, comparing enriched pathways between fish fed CV14 and CD in both the digesta and mucosa^45^.

Mixed effect models, which included a random tank effect, were further employed to evaluate the effect of diet and timepoint (before vs. after stress) on health indicators including plasma stress indicators as well as gene expression in the head kidney and gill. Pairwise contrasts were performed by comparison of least square means under lsmeans^52^ with correction for multiple comparisons based on Tukey. Relative gene expression values obtained from the multigene expression assays were log2 transformed before statistical analysis. Evaluation of the models followed the procedure described above.

### Ethics statement

The experiment has been approved by the Ministry of Agriculture, Rural Areas, European Affairs and Consumer Protection (MLLEV, Kiel, Germany) under project number V 244 – 86776/2021. All guidelines of the EU Directive 2010/63/EU for animal experiments and the national regulations for animal welfare (TierSchVersV) were followed. The experiment was conducted in compliance with the ARRIVE guidelines.

## Results

### Fish performance and proximate body composition

Growth was not the primary response tested in this experiment but despite restricted feeding, growth in terms of SGR and TGC was impaired in fish fed CV14 resulting from an increased FCR (Table 2; *F(3,8)* = *10.37, p* = *0.004*). FCR, SGR and TGC did not differ among the other diet treatments. PER was significantly influenced by the diet F*(3,8)* = *10.74*, *p* = *0.004*, where CV14 had a significantly lower PER than CV2 and CV14w. The diet furthermore influenced PRE (*F(3,8)* = *6.81, p* = *0.014*) and feeding CV2 and CV14w improved PRE compared to the CV14 group. Fish fed CV14 showed significantly higher whole-body ash content than all other treatments (*F(3,8)* = *24.07, p* < *0.001*) and exhibited the lowest energy, fat and protein content among all diets, albeit not significant (Table 2). Condition factor, hepatosomatic and spleen somatic index were not influenced by the diet (Table 2).

**Table 2:** Growth performance, organ indices and proximate composition (wet matter) of Atlantic salmon after eight weeks of feeding the experimental diets.

	CD	CV2	CV14	CV14w	ANOVA
IBW [g]	125.5 ± 2.1	126 ± 3.1	125.7 ± 2	127.3 ± 1.3	0.941
FBW [g]	199.7 ± 2.4	202.7 ± 5.7	191 ± 2.3	205.7 ± 3	0.088
SGR	0.83 ± 0.01^a^	0.85 ± 0.01^a^	0.75 ± 0.01^b^	0.86 ± 0.03^a^	0.003
TGC	0.58 ± 0.01^a^	0.6 ± 0.02^ab^	0.51 ± 0^b^	0.62 ± 0.02^ab^	0.006
FCR	1.24 ± 0.03^a^	1.21 ± 0.01^a^	1.39 ± 0.02^b^	1.21 ± 0.04^a^	0.004
PER	1.75 ± 0.04^ab^	1.87 ± 0.02^b^	1.61 ± 0.02^a^	1.88 ± 0.06^b^	0.004
PRE	32.52 ± 0.47^ab^	34.25 ± 0.58^b^	30.14 ± 0.71^a^	35.13 ± 1.34^b^	0.014
CF	0.93 ± 0.02	0.89 ± 0.02	0.89 ± 0.01	0.89 ± 0.01	0.395
HSI [%]	1.30 ± 0.04	1.21 ± 0.03	1.29 ± 0.02	1.24 ± 0.03	0.218
SSI [%]	0.10 ± 0.01	0.08 ± 0.01	0.09 ± 0.01	0.10 ± 0.01	0.386
Survival [%]	100 ± 0	100 ± 0	100 ± 0	100 ± 0	–
Body composition [% WM]					
Ash	1.94 ± 0.02^a^	1.99 ± 0.03^a^	2.21 ± 0.01^b^	2 ± 0.03^a^	< 0.001
Crude protein	17.88 ± 0.12	17.79 ± 0.06	17.91 ± 0.08	17.93 ± 0.09	0.713
Crude lipid	10.48 ± 0.11	10.66 ± 0.45	9.82 ± 0.23	10.05 ± 0.21	0.206
Energy [MJ/kg WM]	8.29 ± 0.07	8.35 ± 0.21	8.08 ± 0.1	8.17 ± 0.07	0.457

Data is displayed as mean ± SEM, with n = 3 tanks per treatment for performance parameters and proximate body composition and n = 12 individuals for organ specific indices. A significant difference (p < 0.05) among the different diet groups is indicated by different superscript letters and based on Tukey´s multiple comparisons. *IBW* initial body weight, *FBW* final body weight, *SGR* specific growth rate, *TGC* thermal growth coefficient, *FCR* feed conversion ratio, *PER* protein efficiency ratio, *PRE* protein retention efficiency, *CF* Fulton´s condition factor, *HSI* hepatosomatic index, *SSI* spleen somatic index, *WM* wet matter.

### Intestinal microbiota in response to the diet

**Fig. 1:**
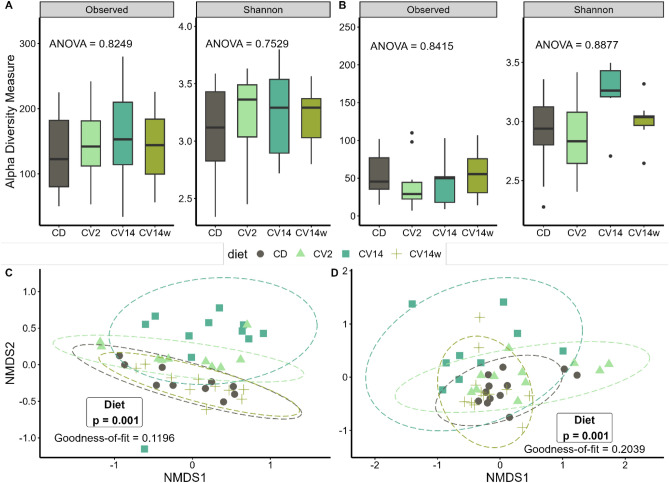
Alpha and beta diversity of the digesta and intestinal mucosa of the hind-gut of Atlantic salmon fed with *C. vulgaris* enriched diets for eight weeks. The salmon received either a control diet (CD), a diet with 2% (CV2) or 14% *C. vulgaris* (CV14) on a daily basis, or a diet containing 14% *C. vulgaris* once weekly. Observed ASVs and Shannon diversity index of digesta (**A**) and mucosa (**B**) do not differ among diets tested by one-way ANOVA (displayed p-value). Non-metric multidimensional scaling on Bray–Curtis distances based on weighed genus-level data revealed significant differences between dietary treatments in both digesta (**C**) und intestinal mucosa (**D**) according to PERMANOVA; n = 10–11 per diet.

A total of 50 digesta and 51 mucosa samples (including initial samples) were obtained after downstream processing. The average read count for digesta samples was 19,850 reads per sample, while it was 3660 reads per sample for mucosa samples. Alpha-diversity (observed and Shannon Diversity) was higher in digesta than in mucosa samples (Wilcoxon, p < 0.001; Fig. 1A,B) but in both sample types, not influenced by the diet (Fig. 1A,B). Beta-diversity, displayed as phylogenetically weighed Bray–Curtis distances using non-metric multi-dimensional scaling (NMDS) indicated a structure based on diet in both digesta (Fig. 1C) and mucosa samples (Fig. 1D). Permutational analysis of variance (PERMANOVA) revealed for both sample types a significant effect of diet (*p* = 0.001*;* Table S2). Beta diversity in the digesta of fish fed CV14 was significantly different from CD and CV14w (*p* = 0.001; Table S2). Similarly, beta diversity in the mucosa of fish fed CV14 was significantly different to fish fed CD and CV14w (*p* = 0.001), as well as to CV2 (*p* < 0.05). Furthermore, microbial beta diversity in both the digesta and mucosa was significantly different between initial samples and samples taken after feeding the experimental diets (Fig. S2; PERMANOVA, *p* = 0.001 for digesta and *p* = 0.003 for mucosa).

**Fig. 2:**
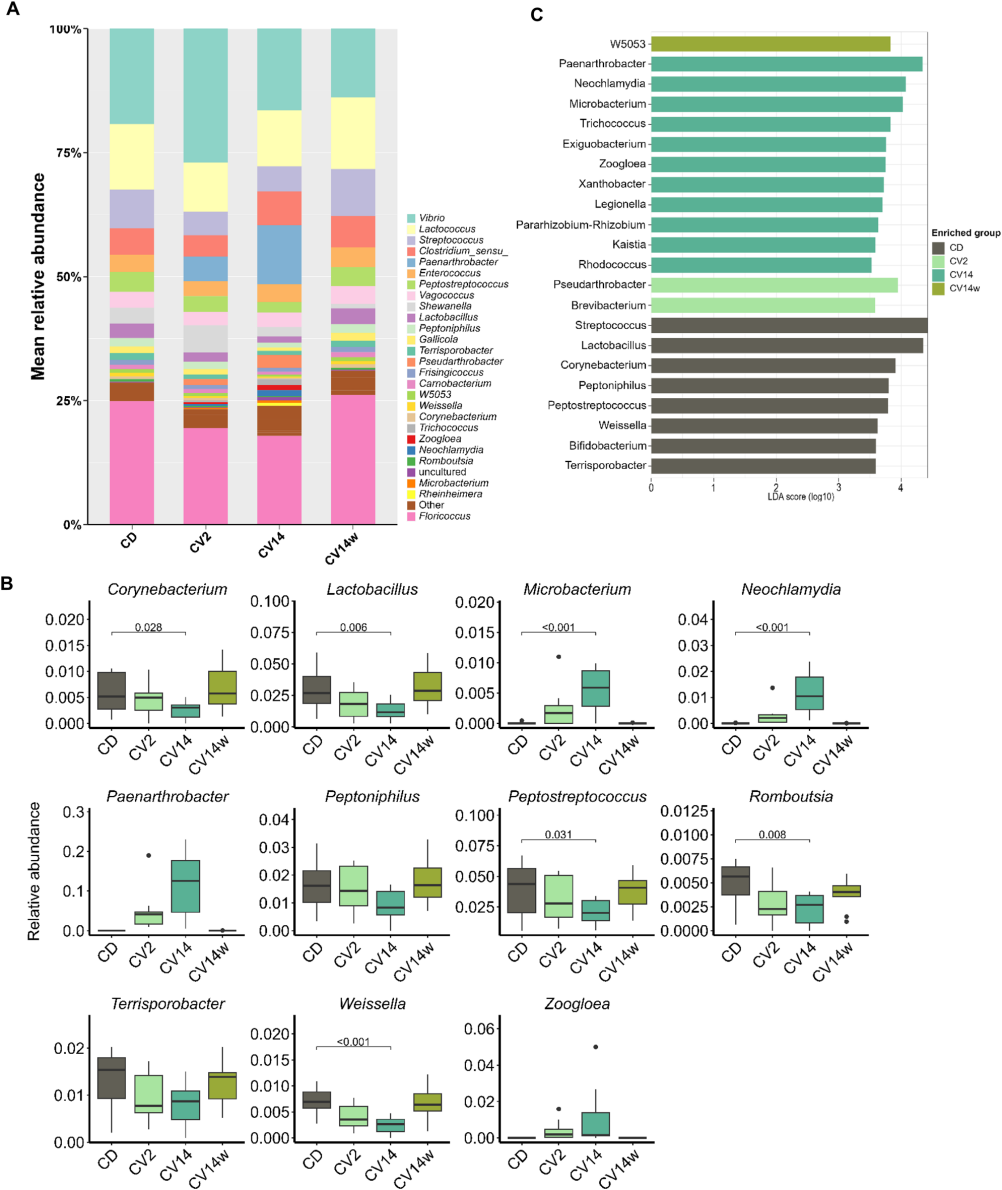
Microbial composition of the digesta of Atlantic salmon fed diets enriched with *C. vulgaris* for eight weeks. The salmon received either a control diet (CD), a diet with 2% (CV2) or 14% *C. vulgaris* (CV14) on a daily basis, or a diet containing 14% *C. vulgaris* once weekly. Mean relative abundance of microbiota on genus level per diet are displayed in a stacked bar plot (**A**). The order of the bars is based on abundance, except for the most abundant phyla, which are placed at the bottom for improved readability. Note that category ‘Other’ includes taxonomical clades with an overall abundance of < 0.5%. Boxplots display relative abundance of main genera between the different diets (**B**). Significantly enriched genera revealed by LEfSe analysis (**C**). Statistically significant differences in panel B between CD and the other diets are represented by the respective p-value (< 0.05); n = 10–11 per diet.

The bacterial community structure of the digesta was comprised of multiple taxa and no single taxa dominated overall (Fig. 2A). However, phyla that were most abundant in the digesta were *Firmicutes*, *Proteobacteria* and *Actinobacteria* (Fig. S3). On the genus level, the most abundant taxa in the digesta were *Floricoccus* (21.8% relative abundance), *Vibrio* (19%), *Lactococcus* (12.1%) and *Streptococccus* (7%; Fig. 2A).

In the digesta, several taxa were enriched in fish fed CV14 compared to the control (CD) diet (Fig. 2B). These included *Microbacterium* (*p* < 0.001) and *Neochlamydia* (*p* < 0.001), as well as *Paenarthrobacter* and *Trichococcus* (Fig. 2B), which were only present in CV2 and CV14. In contrast, *Peptostreptococcus* (*p* = 0.03), *Weissella* (*p* < 0.001), *Corynebacterium* (*p* = 0.03), *Romboutsia* (*p* = 0.008) and *Lactobacillus* (*p* = 0.006) were reduced in fish fed CV14 compared to CD. Linear discriminant analysis of effect size (LEfSe) analysis furthermore revealed that *Paenarthrobacter* was significantly associated with the CV14 diet (LDA score > 4.0), whereas *Streptococcus* and *Lactobacillus* with the control diet (CD; Fig. 2C).

**Fig. 3:**
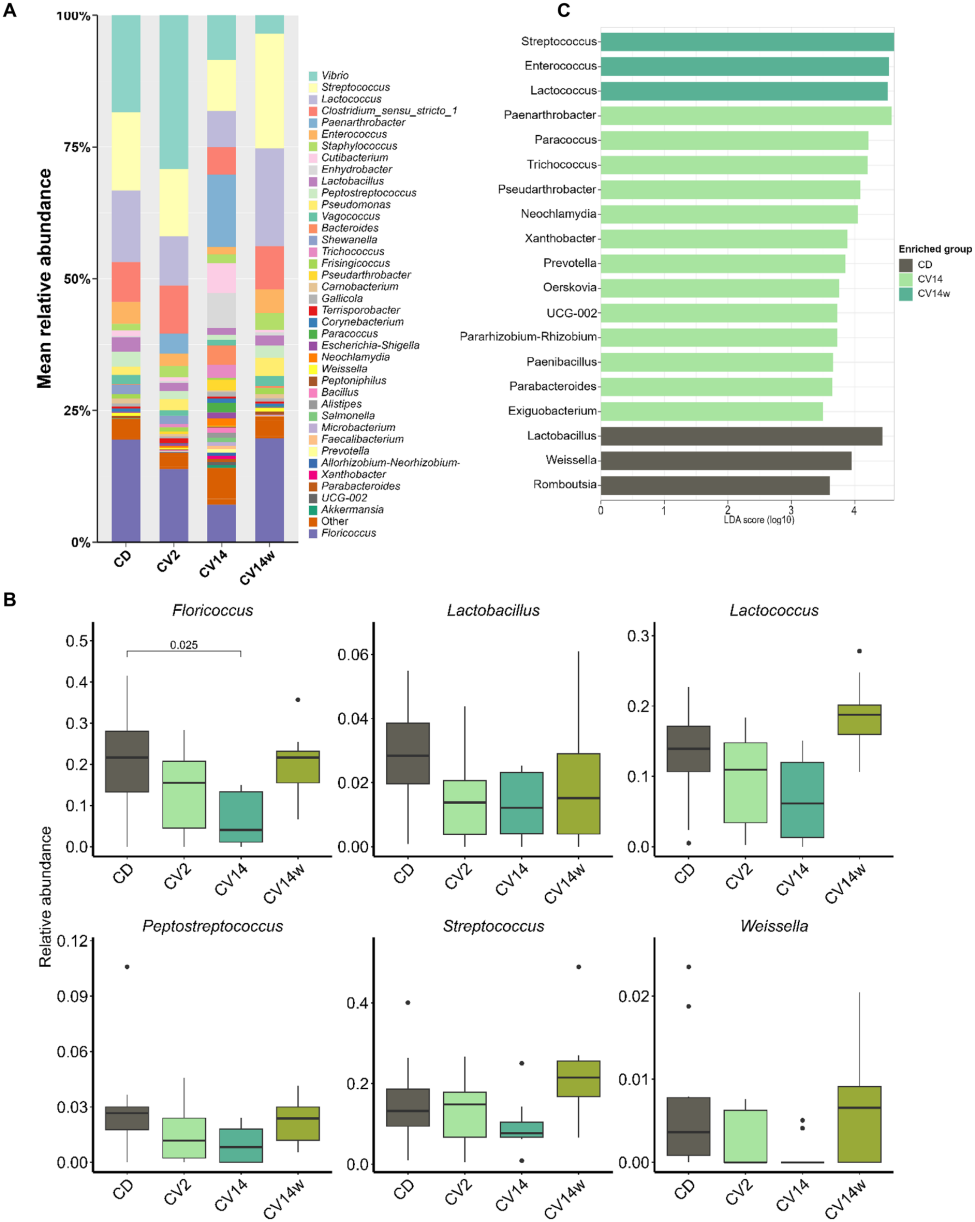
Microbial composition of the intestinal mucosa of Atlantic salmon fed diets enriched with *C. vulgaris* for eight weeks. The salmon received either a control diet (CD), a diet with 2% (CV2) or 14% *C. vulgaris* (CV14) on a daily basis, or a diet containing 14% *C. vulgaris* once weekly (CV14w). Mean relative abundance of microbiota on genus level per diet are displayed in a stacked bar plot (**A**).The order of the bars is based on abundance, except for the most abundant phyla, which are placed at the bottom for improved readability. Note that category ‘Other’ includes taxonomical clades with an overall abundance of < 0.5%. Boxplots display relative abundance of main genera between the different diets (**B**). Significantly enriched genera revealed by LEfSe analysis (**C**). Statistically significant differences in panel B between CD and the other diets are represented by the respective p-value (< 0.05); n = 10–11 per diet.

Similar to the digesta, the majority of bacteria in the intestinal mucosa belong to either *Firmicutes*, *Proteobacteria* or *Actinobacteria* (Fig. S4). The genus *Vibrio* (15%) and *Floricoccus* (15.6%) were the two most abundant across all diets in the mucosa (Fig. 3A). Interestingly, we did not detect the genus *Mycoplasma* in the mucosa samples. *Mycoplasma* was also not detected in initial samples of intestinal mucosa and digesta, during which the fish received a standard commercial diet (Fig. S5).

In the mucosa *Neochlamydia*, *Paenarthrobacter* and *Trichococcus* were enriched in fish fed CV14 compared to CD, where they were not detected (Fig. 3A), while *Floricoccus* was reduced (*p* = 0.025; Fig. 3B). These results were furthermore reflected in the LEfSe analysis, which revealed that, similar to the results for the digesta, *Paenarthrobacte*r in the mucosa was associated with feeding CV14, whereas *Lactobacillus* was associated with the control diet (Fig. 3C).

A total of 152 genera were identified as core microbiota shared between digesta and mucosa samples of the intestine. These included *Vibrio*, *Floricoccus*, *Streptococcus* and *Lactococcus*.

To gain a deeper understanding of potential microbial pathways that were enriched in fish fed CV14 as compared to CD, we conducted PICRUSt2 analysis based on MetaCyc metabolic pathway information for microbiota in the digesta and mucosa. Several predicted microbial pathways were enriched in the digesta of fish fed CV14 compared to CD (Fig. S6). These included the pathways aerobic respiration (cytochrome c), TCA cycle and glucose, xylose and inositol degradation (Fig. S6). In the mucosa pathways associated with nitrogen metabolism such as purine degradation and adenine salvage, as well as biosynthesis pathways of adenosine and guanosine, were enriched in fish fed CD (Fig. S7). Pathways associated with aerobic respiration I and TCA cycle were particularly enriched in fish fed CV14.

### Plasma stress indicators in response to oxidative stress

**Fig. 4:**
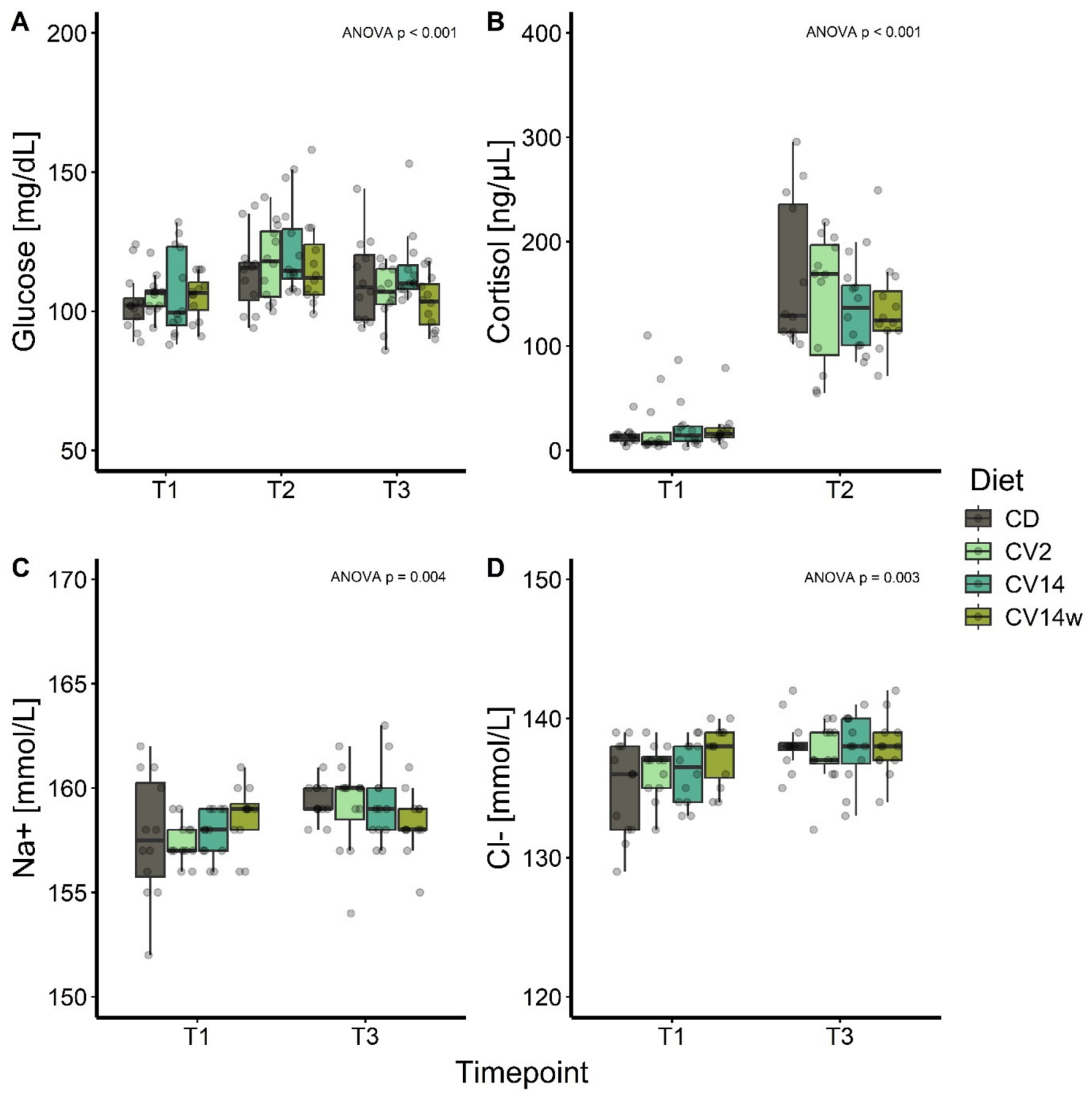
Plasma glucose (**A**), cortisol (**B**), sodium (**C**) and chloride ions (**D**) of Atlantic salmon fed with *C. vulgaris* enriched diets for eight weeks and sampled before (T1), 1 h after (T2) and 18 h after (T3) exposure to peracetic acid-based disinfectant. The salmon received either a control diet (CD), a diet with 2% (CV2) or 14% *C. vulgaris* (CV14) on a daily basis, or a diet containing 14% *C. vulgaris* once weekly (CV14w). Data is presented as boxplots with median, n = 12. Statistical significance (p < 0.05) was assessed by Tukey multiple comparisons and the overall ANOVA timepoint effect for each parameter is displayed in the top right of each panel.

The stress response was investigated regarding classical blood plasma indicators such as glucose and cortisol, as well as the primary ions of the electrolytes sodium (Na^+^) and chloride (Cl^−^). Glucose levels were significantly altered across timepoints (*F(2, 124)* = *12.34, p* < 0.001) and slightly increased one hour after the introduction of PAA (T2) as observed for all treatments with no diet-specific effects (Fig. 4A). After 18 h (T3), the glucose concentrations returned to their initial values. Cortisol increased following exposure to PAA (*F(1, 80)* = *214.83, p* < 0.001) and was not different among diets 1 h after stress exposure (Fig. 4B). An overall increase in sodium (*F(1, 80)* = *8.70, p* = 0.004) and chloride (*F(1, 80)* = *9.71, p* = 0.003) concentrations in the plasma 18 h (T3) after introduction of PAA was detected (Fig. 4 C,D).

### Protein concentration in response to diet and stress

**Fig. 5:**
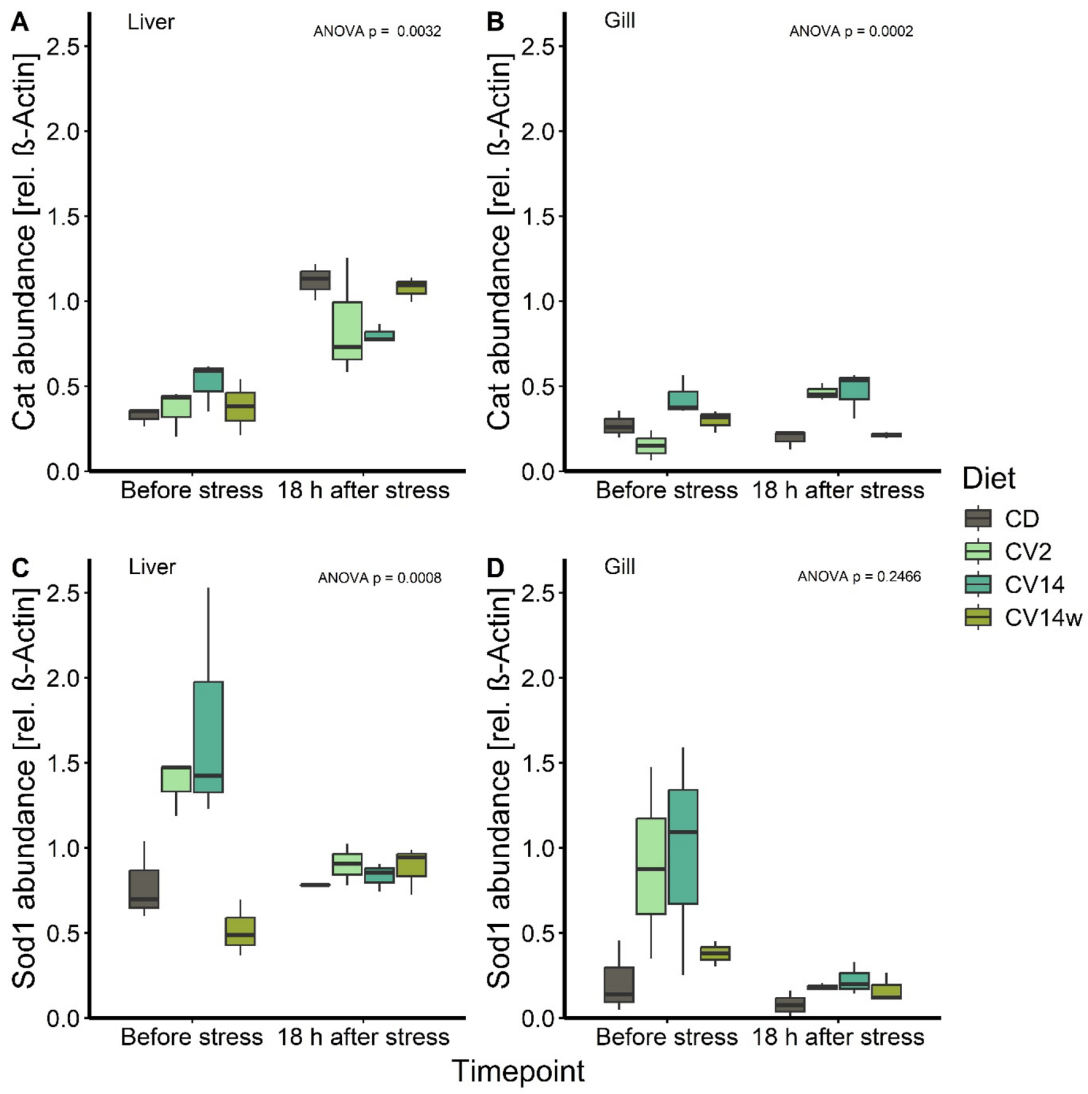
Protein abundance of catalase (Cat) in liver (**A**) and gill (**B**), as well as superoxide-dismutase 1 (Sod1) in liver (**C**) and gill (**D**) of Atlantic salmon fed with *C. vulgaris* enriched diets for eight weeks. The salmon received either a control diet (CD), a diet with 2% (CV2) or 14% *C. vulgaris* (CV14) on a daily basis, or a diet containing 14% *C. vulgaris* once weekly (CV14w). The fish were subsequently exposed to an oxidative stressor via treatment with peracetic acid-based disinfectant and sampled before and 18 h after stress treatment. Samples per organ and diet group represent a pool of four fish per tank (n = 3 tanks) and data is presented as boxplots with median. Statistical significance (p < 0.05) was assessed by multiple comparisons for heterogenous variances and p-values for the ANOVA interaction effect of diet and timepoint are shown in the top right of each panel.

Catalase protein concentrations in the liver increased by threefold 18 h after exposure to PAA in the CD (Tukey; *p* = 0.019; Fig. 5A) and CV14w group (*p* = 0.05). In fish fed CV14, this increase was only modest (Tukey, *p* = 0.21). In the gills, no major changes occurred, except for fish fed CV2 where Cat increased significantly after PAA exposure (Tukey; *p* = 0.001; Fig. 5B) and was significantly higher compared to CV14w (Tukey; *p* = 0.04). The concentration of Sod1 in the liver of fish fed CV14w was only one third of fish fed CV2 before stress (Tukey; *p* = 0.034; Fig. 5C) and decreased in CV2 and CV14 after stress exposure, albeit not significant. Sod1 protein concentrations in the gill were slightly increased before stress in CV2 and CV14. Sod1 levels were reduced after stress exposure in all groups (Fig. 5D) but this reduction was not significant.

### Gene expression in response to oxidative stress

When comparing immune- and stress-regulated gene expression, no significant differences between the different diets within one timepoint nor an overall diet effect was detected (Tables S3, S4). The only exception is an overall diet effect for *ier2-2*, with significantly induced transcript levels (*p* = 0.04) in the head kidney of fish fed CV14w compared to fish fed CD (Table S3). Overall, PAA treatment affected the expression of a large proportion of selected genes involved in different immune- and stress-related pathways (17/44 genes in the head kidney and 24/44 genes in the gill), as indicated by a significant timepoint effect (Table S3, S4). Additionally, significant interactions in the model indicate that the diet influenced the response to the oxidative stressor in both head kidney and gill (Tables S3, S4).

**Fig. 6:**
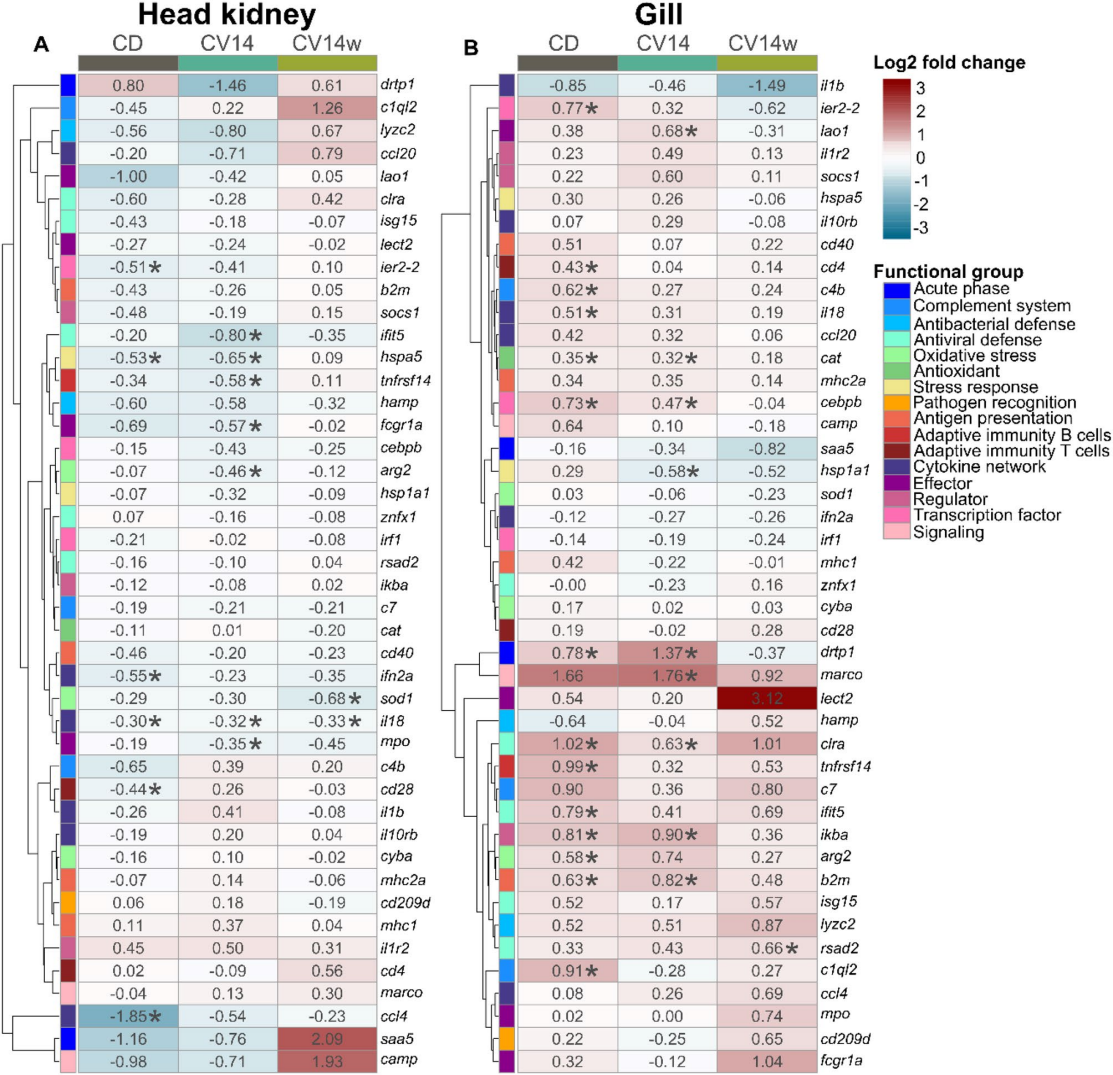
Hierarchical clustering of log2 fold changes of immune- and stress regulated genes in the head kidney (**A**) and gill (**B**) of Atlantic salmon fed with *C. vulgaris* enriched diets for eight weeks, sampled after exposure to oxidative stress and relative to initial gene expression values of selected genes based on their relative transcript levels. Expression of selected genes was studied with quantitative PCR (qPCR). Statistical significance compared to before stress values within the corresponding diet group was assessed by pairwise contrasts (*p < 0.05). The rows represent different genes categorized into functional groups as illustrated in the legend on the right. The columns display fish fed control diet (CD), a diet with 14% *C. vulgaris* (CV14) on a daily basis, or a diet containing 14% *C. vulgaris* once weekly (CV14w). Each cell is colored based on the fold change of that gene, as visualized in the legend on the right. For both figure panels (**A**,**B**) n = 11–12 per timepoint per diet group.

In the head kidney of fish fed CD, the oxidative stressor PAA had only a minor effect on immune- and stress-related gene expression (Fig. 6A). 18 h after PAA exposure, the transcript levels of five genes (*hspa5, ier2-2, ifn2a*, *il18* and *cd28*) were slightly (− 0.30 to − 0.55 log2 fold) but significantly reduced (Fig. 6A; *p* < 0.05). Additionally, a stronger reduction (− 1.85 log2 fold) was observed for *ccl4* transcript numbers (*p* < 0.05). In comparison to the head kidney, exposure to PAA affected stress- and immune-related gene expression in the gill of fish fed CD more substantially (Fig. 6B). Transcript levels of 14 immune- and stress-related genes were enhanced in the gill (Fig. 6B; *p* < 0.05) including *arg2* (0.58 log2 fold), *b2m* (0.63 log2 fold), *c1ql2* (0.91 log2 fold), *c4b* (0.62 log2 fold), *cat* (0.35 log2 fold), *cd4* (0.43 log2 fold), *cebpb* (0.73 log2 fold), *clra* (1.02 log2 fold), *drtp1* (0.78 log2 fold), *ier2-2* (0.77 log2 fold), *ifit5* (0.79 log2 fold), *ikba* (0.81 log2 fold), *il18* (0.51 log2 fold) and *tnfrsf14* (0.99 log2 fold).

Diets supplemented with *Chlorella vulgaris* had only a modest effect on the PAA-induced expression modulation of selected genes. In the head kidney of fish fed CV14, the transcript concentrations of seven genes were significantly reduced after PAA exposure, including *arg2* (− 0.46 log2 fold), *fcgr1a* (− 0.57 log2 fold), *hspa5* (− 0.65 log2 fold), *ifit5* (− 0.80 log2 fold), *il18* (− 0.32 log2 fold), *mpo* (− 0.35 log2 fold) and *tnfrsf14* (− 0.58 log2 fold) (*p* < 0.05). Fish fed with CV14w had − 0.33 log2 fold reduced transcript levels of *il18* and − 0.68 log2 fold reduced transcript levels of *sod1* (*p* < 0.05) after occurrence of the oxidative stressor. None of the selected genes was significantly induced in the head kidney of salmon from both diet groups upon PAA exposure.

In the gill of fish fed with CV14 and CV14w, the expression of selected genes was modulated only slightly after PAA treatment. In gill of fish fed CV14, the transcript levels of eight genes were significantly induced after the oxidative stressor, including *b2m* (0.82 log2 fold), *cat* (0.32 log2 fold), *cebpb* (0.47 log2 fold), *clra* (0.63 log2 fold), *drtp1* (1.37 log2 fold), *ikba* (0.90 log2 fold), *lao1* (0.68 log2 fold) and *marco* (1.76 log2 fold) (*p* < 0.05). Only *hsp1a1* level was significantly reduced by -0.58 log2 fold after PAA exposure in fish fed with CV14 (*p* < 0.05). In the gill of fish fed with CV14w, only *rsad2* level was significantly induced by 0.66 log2 fold and none of the selected genes were reduced after the oxidative stressor.

## Discussion

In this study we show that the amount of microalgae *Chlorella vulgaris* included in a functional diet as well as the mode of application differently affects feed conversion, the intestinal microbiota and response to an acute oxidative stressor in Atlantic salmon. Our data sheds new light on how a functional diet enriched with microalgae contributes to safeguarding health of Atlantic salmon under challenging conditions in recirculating aquaculture systems.

### Feed conversion is impaired at high *Chlorella* inclusion

Feeding a diet with 14% *C. vulgaris* daily to Atlantic salmon post-smolts increased feed conversion efficiency in our study. Contrasting results were reported for diets containing 10 – 20% *C. vulgaris* that improved feed conversion efficiency of gilthead seabream^53^ and Nile tilapia^54^. However, in rainbow trout, inclusion rates of 5–10% of *Chlorella sorokiniana* did not change feed conversion^55^, which seems to be related to the anatomy and function of the digestive system. Omnivorous and herbivorous fish species are able to digest plant-based material, including complex carbohydrates, much more efficiently than carnivorous fish. Salmon that received a diet containing only 2% *C. vulgaris* daily or 14% once weekly, however, showed no tendency towards reduced feed conversion and protein retention efficiency was even improved. Hence, a threshold in the microalgae inclusion rate beyond which potential positive effects turn into negative effects that impair feed conversion of Atlantic salmon seem to exist. This was further reflected in the proximate body composition, which is in line with our previous findings with an inclusion of 8% *C. vulgaris*^22^, as the inclusion of 14% *C. vulgaris* in this study also increased whole-body ash content. A likely explanation for this phenomenon could be an increased bone-to-flesh ratio. Our experiment lasting 56 days at a feeding rate of 1% was overall too short to evaluate growth effects and future studies investigating the influence of different inclusion levels of *C. vulgaris* should be performed over longer time periods providing feed in excess.

### Microbiota in digesta and mucosa are modulated in response to dietary *Chlorella*

The intestinal microbiome plays an important role in nutrient digestion and was generally found in fish to be less complex than in mammals, dominated by often very few taxa^56^. In case of salmonids, these belong to the phyla *Fusobacteria*, *Proteobacteria* and *Firmicutes*^28,29,57^. The two habitats within the intestine, namely digesta and mucosa, harbour distinct microbial communities. In line with what has been found earlier, our results confirm a significantly lower relative abundance and diversity of bacteria residing within the mucosa as compared to bacteria associated with the digesta of fish^36,58^. However, 152 genera were identified as core microbiota shared between digesta and mucosa samples, less than the sum of unique genera of both tissues. This contrasts previous studies in rainbow trout^41^ and Atlantic salmon^59^ which have revealed a smaller core microbiota and less similar microbiota composition among digesta and mucosa. We can only speculate why we found a generally larger overlap between the microbial composition of digesta and mucosa, but it might be related to the type of experimental feed used in the trial. While almost all other studies in salmonids have used extruded feeds, we used pelletized feeds in our study, which generally differ in their water stability and thus retention time within the intestine compared to extruded feeds. Hence, the physical properties of the digesta could have resulted in a microenvironment, that was overall more similar to that of the mucosa when comparing it to that of extruded feeds. The physical aspect of fish feeds should be considered when investigating dietary effects on the microbiome in future studies.

At the phylum level, the dominance of bacteria belonging to *Firmicutes*, *Proteobacteria* and *Actinobacteria* is in line with what has been previously found in the intestine of salmonids^31,57,58,60^. Strikingly, *Mycoplasma*, a bacterium that has been observed to be particularly enriched in the intestinal mucosa of salmonids was not detected in either the mucosa nor in digesta samples. *Mycoplasma* is thought to be tightly linked to salmonid host-health^27,61^, potentially because of its ability to synthesize the essential amino acid arginine. Our results on the microbial composition of digesta and mucosa at the start of the experiment furthermore confirm that even before the application of the experimental feeds and while the fish were fed a standard commercial type diet, the *Mycoplasma* genus was not present in the intestine of the salmon. Colonization of the intestine with the genus *Mycoplasma* was found to be dependent on the host environment^62^ and the absence of *Mycoplasma* in our study suggests that fish haven’t been exposed to *Mycoplasma* before*.* As the salmon were sourced from a fully closed RAS facility importing sterilized eggs, it seems likely that the fish haven’t been exposed and colonized by *Mycoplasma* earlier in life. It has been further found that the abundance of *Mycoplasma* in the intestine of Atlantic salmon is life-stage dependent and increases later in life in seawater^57^, while it was absent in fish held in freshwater RAS^63^. The overall microbial composition in both digesta and mucosa seems to be more complex than reported in many other studies, however, care must be undertaken in these comparisons, as different DNA extraction, sampling- and sequencing methods introduce different biases.

Diet is a main factor shaping the intestinal microbiota in fish and mammals, as its composition selects for a particular microbial community. In this study diet only influenced beta diversity in digesta and mucosa samples, while alpha diversity remained unaffected. In contrast feeding juvenile Nile tilapia a diet supplemented with 2% *Chlorella vulgaris* resulted in a significant increase in observed species and Shannon diversity^64^. A particular function of the microbiota associated with the digesta is the conversion of complex (indigestible) carbohydrates into short-chain fatty acids. The cell wall of *C. vulgaris* is built up of a multitude of complex cellulose- and chitin-like carbohydrates^65^ and when included in fish diets, can provide a specific substrate for bacteria able to breakdown such complex carbohydrates. Interestingly, 14% inclusion of *Chlorella vulgaris* in this study increased the relative abundance of *Trichococcus* in the digesta and metabolic pathway prediction based on the microbial composition revealed an enrichment of carbohydrate degradation pathways for fish fed this diet. Similar results were found in largemouth bass, where feeding diets containing *Chlorella* as a replacement for fishmeal resulted in a significant enrichment of microbial carbohydrate metabolism^66^. *Trichococcus* species are known for their ability to utilize various carbohydrate sources including cellobiose, sucrose and glucose^67–70^ and their presence is likely related to the availability of complex carbohydrates. Although it could generally be beneficial if the bacterial metabolites from the degradation process are available for the fish, in many cases, these are used as energy substrates by the bacteria themselves. This was likely the case in our study, as feed conversion efficiency in fish fed 14% *C. vulgaris* was impaired. The genus *Paenarthrobacter* was furthermore particularly associated with fish fed *C. vulgaris* in the digesta and mucosa but absent in fish fed the control diet. Although this genus was found to exhibit metabolic capacity to degrade herbicides^71^ and iprodione^72^ it seems unlikely that pesticide contents in the microalgae are the reason for their occurrence. The microalgae were analyzed for a variety of pesticides and cultured in closed bioreactors, minimizing the risk of contamination. It seems more likely that the microalgae provide a specific yet unknown substrate, which is preferably used by *Paenarthrobacter.* Interestingly, *Paenarthrobacter* was found to be associated with growth and carotenoid production in the microalgae *Haematococcus lacustris*^73^ and thus carotenoids from *C. vulgaris* might explain why *Paenarthrobacter* was found exclusively in fish fed these diets. *Lactobacillus*, which is considered a probiotic in fish, was reduced in the digesta of fish fed 14% *C. vulgaris* and slightly reduced in fish fed 2% *C. vulgaris*, which aligns with findings in largemouth bass^66^. *Lactobacillus* was found to protect against the development of bacterial infection in various fish species^74–76^. However, the relative abundance of lactic acid bacteria in the intestine of Atlantic salmon has also been found to increase in diets containing fermented soybean meal^77^. During diet formulation for this experiment, inclusion of *C. vulgaris* was done in exchange for soy protein, which could explain the reduced abundance of lactic acid bacteria and thus reduced abundance of *Lactobacillus* may not be detrimental to host health but rather a consequence of a slightly different diet composition. Bacterial composition between fish fed the control diet and fish receiving a diet containing *C. vulgaris* once weekly was very similar. Such short-term perturbation did not induce any lasting effects on the intestinal microbiota, while it remains an open question to what extent recurring feed changes affect the microbiota in fish.

### Response to acute oxidative stress is slightly modulated by functional diets

Stress activates the release of stress hormones such as glucocorticoids, which then induce physiological changes, allowing fish to cope with the stressor^78,79^. Acute stress affects the immune response mainly by inducing distinct innate immune pathways such as the activation of a Th1 immune response and suppressing components of the adaptive immune response^80^.

In previous studies, treatment with PAA induced a systemic, adaptive stress response, which did not lead to chronic manifestations^13,81^. We also observed a systemic stress response in the present study, reflected by an elevation of cortisol and glucose in the plasma of all diet groups one hour after PAA treatment with a subsequent return to initial values of glucose after 18 h. As an oxidative biocide, PAA induces ROS production, which decreased the activity of the antioxidative enzymes Sod1 and Cat in both the liver and gill of grass carp^82^ and in the muscle, liver and heart tissues of rainbow trout^83^. In our study, PAA treatment increased Cat abundance in the liver, while Sod1 abundance remained unchanged or decreased. The enzymes Sod1 and Cat are closely related to each other, in which Sod1 is the first line of defense against ROS and catalyzes the reaction from superoxide anions O_2_• − to H_2_O_2_ and O_2_. Next, Cat converts H_2_O_2_ to the harmless molecules H_2_O and O_2_^84^. The liver is the main detoxifying and metabolizing organ and since the used PAA solution contains 12% H_2_O_2_, this may have induced specifically Cat in the liver to convert the excess H_2_O_2._ Sod1 abundance was increased prior stress in both liver and gill in response to feeding *C. vulgaris*, indicating enhanced potential to eliminate ROS, in line with findings in rainbow trout^55^ and Nile tilapia^23^. Western blot results however yield rough estimates of protein abundances and should be complemented with other markers to allow for a comprehensive interpretation of the results.

The set of stress and immune regulated genes profiled in both head kidney and gill revealed a subtle effect on the expression in the head kidney and more pronounced effects in the gill, being directly exposed to the oxidative stressor. Following exposure to PAA marker genes indicating the activation of Th1-directed immunity (*il18*; interleukin 18*, ccl4*; chemokine C–C motif ligands 4*)* and the presence of T cells (*cd28*; cluster of differentiation 28) were significantly reduced in the head kidney of fish fed CD, indicating the migration of immune cells, including APCs and CD28-positive T cells, from the head kidney to the periphery. In the gill, *il18* and *cd4* (cluster of differentiation 4) were induced, which suggests the immigration of immune cells, specifically (CD4-positive) T cells, to the gill^86^. A slight innate immune reaction occurred in the gill of fish fed the control diet upon PAA exposure, based on our observation of the induced expression of genes coding for receptors such as Clra (c-type lectin receptor A) and Tnfrsf14 (TNF receptor superfamily member 14) as well as downstream-signalling genes such as *cebpb* and *ikba.* The TNFRSF14 protein is expressed on different cells and upon binding with its ligand, activates the NF-κB pathway, resulting in expression of cell survival genes^87^ and managing T-cell immune responses^88^, suggesting a migration of myeloid cells to the gill. Furthermore *ikba* (encoding for NF-κB inhibitor α) controls the NF-κB transcription factor, which is an important driver of immune and stress responses in salmonid fish^89^. Moreover, in line with the results of the western blots and a study in Atlantic salmon parr^81^, the transcript levels of *cat* (catalase) were enhanced in the gill, suggesting a counteracting reaction in response to PAA. Stress-related genes (*drtp1;* differentially regulated trout protein 1, *ier2-*2; immediate early response 2–2) were increased in the gill, whereas in the head kidney, *ier2-2* and *hspa5* (heat shock protein family A (Hsp70) member 5) were reduced after PAA exposure. The exact functions of *drtp1* and *ier2-2* are not yet fully understood^90^, but like *hspa5,* they seem to respond to external stimuli such as stress. This implies that PAA primarily activates a local stress response in the gill.

One main aspect of the present study was to assess the potential of the microalgae enriched diets to modulate the transcriptional response to PAA. Although the overall response to PAA was similar in fish fed a diet containing 14% *C. vulgaris* daily to the control diet, few genes were specifically up or downregulated following exposure to PAA only in this treatment. In the head kidney PAA treatment significantly reduced the expression of genes involved in immune functions, namely T-cell immune responses (*tnfrsf14*), binding of host antibodies presenting, for example, antigens (*fcgr1a*; Fc gamma receptor Ia^91^), antiviral defense (*ifit5*)^92^, anti-inflammatory response (*arg2*)^93^ and anti-microbial defense (*mpo;* myeloperoxidase)^94^. The reduction of these gene products suggests a slightly reduced immune activity in the head kidney after PAA treatment in fish fed 14% *C. vulgaris* daily, which resulted from the emigration of immune cells. In the gill *marco* and *lao1* (l-amino acid oxidase 1) were significantly enhanced in fish fed with CV14 after PAA exposure. As *marco* encodes a pathogen recognition receptor^95^ and *lao1* encodes an enzyme with antibacterial and antiparasitic activity^96^, the induction of these genes likely relate to the immune-modulating properties of *Chlorella vulgaris,* which has been reported to exert strong antibacterial activity^97–99^. Interestingly, feeding 14% *C. vulgaris* daily seems to interfere with the stress response in the gill, where reduced transcript level of *hsp1a1* (Heat shock 70 kDa protein) and an increased level of *drtp1* after PAA treatment was detected. Both genes respond to a variety of stressors^46,100,101^ and the reduced expression of the heat shock protein may suggest that no significant protection against external damage was necessary when fish were fed 14% *C. vulgaris*.

While differential expression of several selected immune- and stress-related genes was apparent in fish fed the control diet and 14% *C. vulgaris* daily, exposure to PAA had negligible effects on the expression of the investigated genes in fish receiving 14% *C. vulgaris* once weekly. As the observed primary adaptive stress response was similar among all dietary groups, it appears most likely that the absence of a transcriptional response in fish fed 14% *C. vulgaris* once weekly reflects an enhanced resilience against PAA. Before stress a significantly increased expression of *ier2-2* indicates an activated immediate early response, a stress responsive gene that was found to increase after smolts were transferred to seawater^102^ and which was rapidly induced in rainbow trout eggs after irradiation^103^. The antiviral gene *rsad2* was the only gene significantly upregulated in the gill after PAA treatment and increased expression of this interferon stimulated gene may indicate increased potential to reduce viral replication^104^. Overall based on our results and others^13^ the treatment with PAA according to current practice can be considered a rather mild stressor. As such we might not have revealed the full potential of supplementing *C. vulgaris* to influence the immune and stress response of fish.

Stress, reduced welfare and disease cause significant economic damage to Atlantic salmon producers. Hence measures to improve health and welfare such as the use of functional feed ingredients to promote better health are both ethically and economically attractive. Based on this study, weekly supplementation of 14% *C. vulgaris*, as opposed to daily enrichment of 2% or 14% *C. vulgaris*, appears to be the preferred approach to increase the resilience to disinfectant-induced stress. This form of functional supplement application could best be implemented in land-based facilities, where changing among feeds is logistically easier to implement compared to farms at sea. In all cases implementing novel feed ingredients should not compromise the salmon’s growth performance why inclusion levels in the feed for microalgae having a cell wall should be kept low. Although *Chlorella vulgaris* is the most widely and among the most cost-effective produced microalgae, production volume and price currently limit its widespread implementation in the aquafeed industry, making time-restricted e.g. weekly applications particularly attractive.

Overall, this study revealed that the health of Atlantic salmon can be influenced by dietary application of *Chlorella vulgaris* where the intestinal microbiota was most influenced by feeding 14% *C. vulgaris* daily, whereas weekly feeding seemed to increase stress resilience to PAA. As such future studies evaluating functional feed ingredients should not only focus on optimal inclusion rates, but also determine most efficient feeding strategies, optimizing beneficial effects while reducing costs.

## Supplementary Information

Supplementary Information.

## Data Availability

The raw sequences that support the findings of this study are deposited in the sequence read archive (SRA) database from NCBI under the BioProject PRJNA1083322.
