# Supplementary material for: Dietary Chlorella vulgaris supplementation modulates health, microbiota and the response to oxidative stress of Atlantic salmon
Source: Sci Rep. 2024 Oct 10;14:23674. doi: 10.1038/s41598-024-72531-8 (PMC11467335; doi:10.1038/s41598-024-72531-8)
Supplement: Supplementary file 1 — Supplementary Information. [file 41598_2024_72531_MOESM1_ESM.docx]

Supplementary Material

**Can dietary *Chlorella vulgaris* supplementation improve health of Atlantic salmon? Insights from combined analysis of microbiota, host immunity and the response to oxidative stress**

**Tables**

**Table S1.** Genes and their corresponding primers used in this study.

| Gene symbol | Gene | Primer sequence 5’→3’ (sense, antisense) | NCBI-nucleotide accession codes | Primer efficiency (%) | Amplicon length (bp) |
| --- | --- | --- | --- | --- | --- |
| *Acute phase* | | | | | |
| *drtp1* | Differentially regulated protein 1 | ACCTAAAGGAAGTACAATCAGATCA,  ACAACGGTTGCATTTCAGACCC | XM_014150226;  XM_014151528 | 93.7 | 126 |
| *saa5** | Serum amyloid A-5 | TTCCACGCTCGGGGCAACTAT,  AGCTGCTGAGTCCTCATGTCC | NM_001146565 | 93.4 | 129 |
| *Complement system* | | | | | |
| *c1ql2* | C1q-like adipose protein | AGGTAGCCTTCTCAGCTTCACT,  TTCCACTCACCGGCGCCTTAA | XM_014183971; XM_014208680; XM_014187765; XR_001321949 | 98.6 | 92 |
| *c4b* | Complement component C4 | TAGCCTGTACGAAGAGGCAATC,  GAAATTGACATTCTCTCCTGGATT | XM_014162647; XM_014214474 | 83.2 | 187 |
| *c7* | Complement component C7 | CAGGTTCGCACACGGACTGTT, GTCCTGAGGAACAGCGGAACC | XM_014134762 | 98.4 | 148 |
| *hamp** | Hepcidin | GGTGCGAACGGAGGAGGTTG,  ACAGGGAGAGGTGGATCTGAC | XM_014170058; XM_014170044; NM_001140849 | 92.7 | 122 |
| *Antibacterial defense* | | | | | |
| *lyz2* | Lysozyme C II precursor | CTGCAGCCAGCTGCTAACTGA, AAGGCGCCAAGCCACCCATG | XR_001321030; BT057448; BT047934; XM_014145497 | 96.3 | 103 |
| *Antiviral defense* | | | | | |
| *clra* | C type lectin receptor A | ATCTCCAGCAGAGGAAACAAGC,  CCTGCTAAATAAGAGCACGCTCA | BT045294; BT048122; BT046430; NM_001123579 | 98.5 | 162 |
| *ifit5* | Interferon-induced protein with tetratricopeptide repeats 5-2 | ACAAGATGAGCCCTTCGGTCAA, GCCTTGTGGAGATACTCAAGAG | XM_014179290; BT046021; XM_014179291; XM_014158291 | 98.2 | 241 |
| *isg15* | Interferon-stimulated gene 15 | CCTCAACAATATCTACTGAACATATA, CACAGTGGTGTGTGGCTGAACT | NM_001123640; BT049918 | 101.6 | 101 |
| *rsad2* | Radical S-adenosyl methionine domain-containing protein 2; Viperin (rsad) | AGGTGATTTCTTGGGGAAATTAGT, TATTCGCCATACGTTTGGAACCA | BT047340; XM_014186947; NM_001140939 | 100.9 | 120 |
| *znfx1* | Zinc Finger NFX1-Type Containing 1 | AGAGAAGGGCTTTGTCCAGATAA, TACCTCTGGAAGGGCAGATCCT | XM_014168158 | 98.3 | 174 |
| *Oxidative stress* | | | | | |
| *arg2* | Arginase-2 | GACAGAAAAGGAGAGGGGTGGA,  GAAGCTCAGGTCTCCAAAATCAT | XM_014211724; BT058927 | 97.2 | 113 |
| *cyba* | Cytochrome b-245 light chain | TCATTCTCCTTACTGGTGGCATA, CTTGCCCCTTGGGTACTCTAG | XM_014175738; XM_014127044 | 100.8 | 119 |
| *sod1** | Superoxide dismutase 1 | ACCATGGTGATCCATGAGAAGG,  TAGGGCTCCCAGTAAGGGTGT | XM_014198383 | 90.3 | 150 |
| *Antioxidant* | | | | | |
| *cat* | Catalase | TTCTACACTGACGAGGGCAACT, TGGATGAAGGATGGGAACAGCA | BT059457; NM_001140302; XM_014123794; BT049990; XM_014123795; XM_014123797; XM_014173127 | 101.3 | 92 |
| *Stress response* | | | | | |
| *hsp1a1* | Heat shock 70 kDa protein | TAAACTGCAACTAACTCCAAACCA,  TGTCTGTGAAGGCCACATAGCT | XM_014192866; XM_014191271; BT059340; BT046112; XM_014203983; XM_014187508 | 95.1 | 174 |
| *hspa5* | 78 kDa glucose-regulated protein (grp78) | AGTTTGAGGAGCTCAACATGGAC,  TCCTTCACCAGCTGCTGGACC | NM_001141642, XM_014136127 | 103.2 | 154 |
| *Pathogen recognition* | | | | | |
| *cd209d* | CCAAT enhancer binding protein beta | ATCTCTCAGATGGAAGAACAAGAC, TTTAGGGAGATGTTGAGAATGACT | BT048497; NM_001141046 | 97.3 | 94 |
| *Antigen presentation* | | | | | |
| *b2m* | beta-2 microglobulin | GACTTAATGGGGGGCGTTCCA, GGCACCTTGGCACAAAGTGTTA | BT058362; BT048032; AF180487; AF180488; AF180480; AF180485; BT056667; AF180483; BT047559; XM_014184298; NM_001245913 | 101.9 | 120 |
| *cd40* | CD40 (TNFR5) | ACCACACACTAAATGCGGACCA, GTGGGTACTGACACACAGACAAT | BT049620; NM_001141236 | 96.9 | 210 |
| *mhc1* | MHC class Ia heavy chain | AAGGCTTTGTTCCGGCCAGCA, GACGTTCCTCTTCAGCACCTTC | XM_014177344; AF504022; AF504016; EF427384; EF210363; AF504019; AF504021; DQ091798; JN561333; AF504020; AF504015; AF504024; AF504018; AF504013; AF504014; AF504017; AF508864; DQ091795; L07605 | 96.4 | 123 |
| *mhc2a* | HLA class II histantigen, DP alpha chain | ATCTGGGAGCCTGAGGTGATC, GATGGTGAGGTCCTCTGTATTTAT | XM_014133066 | 99.5 | 187 |
| *Adaptive immunity B cells* | | | | | |
| *tnfrsf14* | TNF receptor superfamily member 14 | GCCAGAAATATGGGAGGTTTTGA, CCTCAGTCCTCACCAAGTCAC | XM_014147236 | 100.1 | 117 |
| *Adaptive immunity T cells* | | | | | |
| *cd28* | CD28 | TGAAGCTCTCCAGTGCCAGGC, AGGCGATGACAGTGACAGCCA | XM_014151277 | 99.6 | 134 |
| *cd4* | CD4 | CAGTCAATGTCCACCCCTCCAA, CCTGTGTCTGACAGGGCCACA | XM_014200556 ; XM_014200553 ; XM_014200557 ; XM_014200554 ; XM_014200552 ; XR_001328802; XM_014163618 ; NM_001128603 | 94.9 | 187 |
| *Cytokine network* | | | | | |
| *ccl20* | C-C motif chemokine 20-like | GTTCAACCTCAGCTGCATACGG, GCTCTGTAGTTTGTAGCCTTTTAT | XM_014191476; BT125488; BT057039; BT047144 | 96.0 | 104 |
| *ccl4* | CC chemokine 4 | ACCACCGAACATCTCTCTAGCA, GTGAACCTTGTTTGCATCAGTGT | XM_014191808 | 99.8 | 118 |
| *ifn2a* | Interferon alpha | AAAAATGAACTACAGTGCACAGGC, AAACTGGTAAGGGCGTAGCTTC | NM_001123710; NM_001123570; XM_014204440; XM_014192596; XM_014187639; XM_014192595 | 101.1 | 137 |
| *il10rb* | Interleukin-10 receptor subunit beta-like | AGTTTATCATGGATGAACACACCA, CCAGTAGGTGATGTTAAAGGTTG | XM_014174580; XM_014163988; BT059022; BT047745; XM_014174581 | 104.9 | 155 |
| *il18* | Interleukin-18 | CGGAGGTACCACACATGAATAG, GTCACTCGTAAATGTCGCCTTC | XM_014162473; BT125392; BT057200; NM_001141408; XM_014162472; XM_014162471 | 110.3 | 135 |
| *il1b* | Interleukin-1 beta | CATCATCGCCATGGAGAGGTTA, CAACTCCAACACTATATGTTCTTC | NM_001123582; XM_014143360 | 95.8 | 124 |
| *Effector* | | | | | |
| *fcgr1a* | Ig Fc receptor I | AAATAGAGGTGGACGGTGGGTG, GAAGCTCCACCCCGTCTTTGT | XM_014162559; BT058951; BT058413; XM_014162558; BT058649; XM_014214561 | 97.1 | 144 |
| *lao1* | L-amino-acid oxidase | GGCACAAGGTGACTTTAATAGAG, AAAAGAATTTTGTGAAAGCTAGGGA | XM_014213791; AB831259; XM_014213790 | 101.8 | 130 |
| *lect2* | Leukocyte cell-derived chemotaxin 2-1 | GTGGAGAGGGTCTGTGCTTTAA, TGATCCCTGGGTAGACACTTTG | XM_014211962; XM_014199364; BT059281; XM_014211966 | 98.1 | 120 |
| *mpo* | Myeloperoxidase | GAAAAGAGTCTGGAGAGAGTGC, GACTTTCTCATGGATCAGTCTCA | XM_014131319; XM_014147274; XM_014147173; XM_014128513 | 101.4 | 147 |
| *Regulator* | | | | | |
| *ikba* | NF-kappa-B inhibitor alpha | GCTAGTGGACGACAGTGGCAA, TTCTGACCACTGTAGTTTGGTGT | BT043997; BT058522; BT048185; BT048105; XM_014211117; BT125370; BT072741; XM_014193164 | 100.7 | 144 |
| *il1r2* | IL-1 receptor type II | CCAAGCAAACTGACATTGAGAAG, TGGAGTCCTTATACCACTCTATTT | NM_001145420 | 98.6 | 129 |
| *socs1* | Suppressor of cytokine signaling 1 | CACAGTCGACTGAAGGACGTC, TCACTTCCTGCCAGGCTGAAC | XM_014187410; XM_014190655 | 96.0 | 155 |
| *Transcription factor* | | | | | |
| *cebpb* | CCAAT enhancer binding protein beta | ACGACAGGGATTGCTTTGCTTTC, CCTCTCTGCTGAGTTTCGTTATG | BT049364; BT058424; XM_014146703; EU668996; NM_001139913 | 91.4 | 208 |
| *ier2-2* | Immediate early response 2-2 | ATACAGCACGACACTGATAACATA, TCAGTACAGTTTCTGGCACAATC | NM_001140121; XM_014181789 | 100.5 | 159 |
| *irf-1* | Interferon regulatory factor1 | GGGCCATGCACACAGGGAAAT, GCTTTTGTCTTTCACTTCCTCTAT | NM_001252361; XM_014136758 | 96.2 | 122 |
| *Signaling* | | | | | |
| *camp* | Cathelicidin | GGGGCAGCAAGGCCAACCAT, CCGGTCAGTGTTGAGGGTGTT | XM_014190921; NM_001123573; XM_014140492; XM_014140493 | 103.0 | 112 |
| *marco* | Macrophage receptor | TACACCTTCCAGAGCAGCGATC, CGAAAAGACTTTGTAAAGGAGGAA | XM_014164760; XM_014164759; XM_014173984; XM_014164758 | 99.8 | 129 |
| *Reference genes* | | | | | |
| *rpl4* | Ribosomal Protein L4 | CACCGCATTGAGGAGATCCCA, ATGTCGTTCCAGGCCTTGAGTT | NM_001141783.1 | 94.6 | 116 |
| *rps20* | Ribosomal Protein S20 | GCAGACCTTATCCGTGGAGCTA, TGGTGATGCGCAGAGTCTTG | PMC3031229 | 101.0 | 85 |
| *actb* | Actin Beta | CCAAAGCCAACAGGGAGAAG, AGGGACAACACTGCCTGGAT | PMC3031230 | 100.3 | 91 |

Table S2. PERMANOVA results for the effect of diet on the intestinal digesta and mucus microbiota beta diversity as well pairwise comparisons using permutation MANOVAs on a distance matrix.

|  | PERMANOVA | | | Pairwise comparisons (p- value) | | | | | |
| --- | --- | --- | --- | --- | --- | --- | --- | --- | --- |
|  | **F** | **R^2^** | **Pr(>F)** | **CD - CV2** | **CD - CV14** | **CD - CV14w** | **CV2 - CV14** | **CV2 - CV14W** | **CV14 - CV14w** |
| Digesta | 2.504 | 0.169 | 0.002 | 0.162 | 0.001 | 0.795 | 0.096 | 0.103 | 0.001 |
| Mucosa | 2.072 | 0.135 | 0.001 | 0.206 | 0.001 | 0.551 | 0.034 | 0.007 | 0.001 |

Table S3. Results of linear mixed-effect models and subsequent multiple comparisons for 44 stress- and immune-regulated genes expressed in the head kidney of Atlantic salmon fed with diets enriched with *C. vulgaris* sampled before and after stress.

|  | ANOVA (p-value) | | | Effect | | |
| --- | --- | --- | --- | --- | --- | --- |
| Gene | **Diet** | **Timepoint** | **Interaction** | **Diet** | **Timepoint** | **Interaction** |
| *arg2* | 0.81 | 0.04 | 0.17 |  | T1>T3 | CV14: T1>T3 |
| *b2m* | 0.03 | 0.05 | 0.31 |  | T1>T3 |  |
| *c1ql2* | 0.46 | 0.32 | 0.24 |  |  |  |
| *c4b* | 0.07 | 0.30 | 0.17 |  |  |  |
| *c7* | 0.81 | 0.03 | 0.88 |  | T1 > T3 |  |
| *camp* | 0.11 | 0.08 | 0.06 |  |  |  |
| *cat* | 0.37 | 0.18 | 0.72 |  |  |  |
| *ccl20* | 0.53 | 0.19 | 0.57 |  |  |  |
| *ccl4* | 0.89 | <0.01 | 0.03 |  | T1 > T3 | CD: T1>T3 |
| *cd209d* | 0.37 | 0.52 | 0.15 |  |  |  |
| *cd28* | 0.34 | 0.36 | <0.01 |  |  | CD: T1>T3 |
| *cd4* | 0.34 | 0.91 | 0.44 |  |  |  |
| *cd40* | 0.02 | 0.01 | 0.90 |  | T1 > T3 |  |
| *cebpb* | 0.46 | 0.04 | 0.67 |  | T1 > T3 |  |
| *clra* | 0.23 | 0.09 | 0.10 |  |  |  |
| *cyba* | 0.27 | 0.41 | 0.57 |  |  |  |
| *drtp1* | 0.76 | 0.21 | 0.24 |  |  |  |
| *fcgr1a* | 0.27 | <0.01 | 0.19 |  | T1 > T3 | CV14: T1>T3 |
| *hamp* | 0.71 | 0.05 | 0.92 |  | T1 > T3 |  |
| *hsp1a1* | 0.46 | 0.40 | 0.69 |  |  |  |
| *hspa5* | 0.15 | <0.01 | <0.01 |  | T1 > T3 | CD, CV14: T1>T3 |
| *ier2-2* | 0.03 | <0.01 | 0.30 | CD < CV14w | T1 > T3 | CD: T1>T3 |
| *ifit5* | 0.80 | 0.01 | 0.37 |  | T1 > T3 | CV14: T1>T3 |
| *ifn2a* | 0.50 | <0.01 | 0.54 |  | T1 > T3 | CD: T1>T3 |
| *ikba* | 0.83 | 0.32 | 0.81 |  |  |  |
| *il10rb* | 0.20 | 0.83 | 0.46 |  |  |  |
| *il18* | 0.05 | <0.01 | 0.93 |  | T1 > T3 | CD, CV14, CV14w: T1>T3 |
| *il1b* | 0.69 | 0.68 | 0.74 |  |  |  |
| *il1r2* | 0.50 | 0.08 | 0.71 |  |  |  |
| *irf1* | 0.05 | 0.09 | 0.74 |  |  |  |
| *isg15* | 0.86 | 0.37 | 0.61 |  |  |  |
| *lao1* | 0.33 | 0.16 | 0.14 |  | T1 > T3 |  |
| *lect2* | 0.14 | 0.23 | 0.78 |  |  |  |
| *lyzc2* | 0.23 | 0.05 | 0.16 |  |  |  |
| *marco* | 0.23 | 0.94 | 0.92 |  |  |  |
| *mhc1* | 0.75 | 0.44 | 0.63 |  |  |  |
| *mhc2a* | 0.04 | 0.95 | 0.97 |  |  |  |
| *mpo* | 0.19 | <0.01 | 0.36 |  | T1 >T3 | CV14: T1>T3 |
| *rsad2* | 0.68 | 0.48 | 0.94 |  |  |  |
| *saa5* | 0.11 | 0.04 | 0.35 |  |  |  |
| *socs1* | 0.70 | 0.14 | 0.09 |  |  |  |
| *sod1* | 0.57 | <0.01 | 0.16 |  | T1 > T3 | CV14w: T1>T3 |
| *tnfrsf14* | 0.09 | 0.04 | 0.06 |  | T1 > T3 | CV14: T1>T3 |
| *znfx1* | 0.94 | 0.49 | 0.61 |  |  |  |

Three experimental treatments compared were enriched with 14% C. vulgaris daily (CV14), 14% C. vulgaris weekly (CV14w) and one control diet (CD). Overall model results of the linear mixed effect models with diet and timepoint as fixed factors and tank as a random factor are given in the first three columns. In the last two additional columns, the direction of the effect is displayed, based on the subsequent multiple contrast test performed. Note that although there was an overall diet effect stated, no effect was specified as there was no significant difference in the microalgae fed groups as compared to the control; n = 11 - 12 per diet and timepoint.

Table S4. Results of linear mixed-effect models and subsequent multiple comparisons for 44 stress- and immune-regulated genes expressed in the gill of Atlantic salmon fed with diets enriched with *C. vulgaris* sampled before and after stress.

|  | ANOVA (p-value) | | | Effect | | | |
| --- | --- | --- | --- | --- | --- | --- | --- |
| Gene | **Diet** | **Timepoint** | **Interaction** | | **Diet** | **Timepoint** | **Interaction** |
| *arg2* | 0.88 | <0.01 | 0.29 | |  | T1 < T3 | CD, CV14: T1 < T3 |
| *b2m* | 0.19 | <0.01 | 0.60 | |  | T1 < T3 | CD, CV14: T1 < T3 |
| *c1ql2* | 0.72 | 0.06 | 0.01 | |  | T1 < T3 | CD: T1 < T3 |
| *c4b* | 0.46 | <0.01 | 0.15 | |  | T1 < T3 | CD: T1 < T3 |
| *c7* | 0.44 | 0.01 | 0.60 | |  | T1 < T3 |  |
| *camp* | 0.95 | 0.04 | 0.32 | |  | T1 < T3 |  |
| *cat* | 0.73 | <0.01 | 0.55 | |  | T1 < T3 | CD, CV14: T1 < T3 |
| *ccl20* | 0.44 | 0.01 | 0.74 | |  | T1 < T3 |  |
| *ccl4* | 0.48 | 0.18 | 0.57 | |  |  |  |
| *cd209d* | 0.40 | 0.83 | 0.85 | |  |  |  |
| *cd28* | 0.58 | 0.06 | 0.36 | |  |  |  |
| *cd4* | 0.92 | 0.01 | 0.71 | |  | T1 < T3 | CD: T1 < T3 |
| *cd40* | 0.28 | 0.07 | 0.44 | |  | T1 < T3 |  |
| *cebpb* | 0.63 | <0.01 | 0.12 | |  | T1 < T3 | CD, CV14: T1 < T3 |
| *clra* | 0.19 | <0.01 | 0.88 | |  | T1 < T3 | CD, CV14: T1 < T3 |
| *cyba* | 0.81 | 0.64 | 0.74 | |  |  |  |
| *drtp1* | 0.75 | <0.01 | 0.06 | |  | T1 < T3 | CD, CV14: T1 < T3 |
| *fcgr1a* | 0.95 | 0.30 | 0.39 | |  |  |  |
| *hamp* | 0.68 | 0.76 | 0.40 | |  |  |  |
| *hsp1a1* | 0.37 | 0.08 | 0.01 | |  |  | CV14: T1 > T3 |
| *hspa5* | 0.62 | 0.67 | 0.37 | |  |  |  |
| *ier2-2* | 0.35 | <0.01 | 0.11 | |  | T1 < T3 | CD: T1 < T3 |
| *ifit5* | 0.68 | <0.01 | 0.55 | |  | T1 < T3 | CD: T1 < T3 |
| *ifn2a* | 0.14 | 0.26 | 0.87 | |  |  |  |
| *ikba* | 0.29 | <0.01 | 0.24 | |  | T1 < T3 | CD, CV14: T1 < T3 |
| *il10rb* | 0.43 | 0.69 | 0.35 | |  |  |  |
| *il18* | 0.91 | <0.01 | 0.48 | |  | T1 < T3 | CD: T1 < T3 |
| *il1b* | 0.46 | 0.13 | 0.76 | |  |  |  |
| *il1r2* | 0.35 | 0.24 | 0.52 | |  |  |  |
| *irf1* | 0.65 | 0.01 | 0.90 | |  | T1 > T3 |  |
| *isg15* | 0.99 | 0.01 | 0.55 | |  | T1 < T3 |  |
| *lao1* | 0.66 | 0.01 | 0.09 | |  |  | CV14: T1 < T3 |
| *lect2* | 0.76 | 0.01 | 0.87 | |  | T1 < T3 |  |
| *lyzc2* | 0.98 | <0.01 | 0.87 | |  | T1 < T3 |  |
| *marco* | 0.63 | <0.01 | 0.43 | |  | T1 < T3 | CV14: T1 < T3 |
| *mhc1* | 0.79 | 0.46 | 0.18 | |  |  |  |
| *mhc2a* | 0.80 | 0.01 | 0.94 | |  | T1 < T3 |  |
| *mpo* | 0.96 | 0.17 | 0.91 | |  |  |  |
| *rsad2* | 0.90 | <0.01 | 0.65 | |  | T1 < T3 | CV14w: T1 < T3 |
| *saa5* | 0.65 | 0.22 | 0.64 | |  |  |  |
| *socs1* | 0.34 | 0.07 | 0.38 | |  | T1 < T3 |  |
| *sod1* | 0.81 | 0.15 | 0.30 | |  |  |  |
| *tnfrsf14* | 0.37 | <0.01 | 0.34 | |  | T1 < T3 | CD: T1 < T3 |
| *znfx1* | 0.99 | 0.75 | 0.18 | |  |  |  |

Overall model results of the linear mixed effect models with diet and timepoint as fixed factors and tank as a random factor are given in the first three columns. In the last two additional columns, the direction of the effect is displayed, based on the subsequent multiple contrast test performed. Note that although there was an overall diet effect stated, no effect was specified when there was no significant difference in the microalgae fed groups as compared to the control; n = 11 - 12 per diet and timepoint.

**Figures**


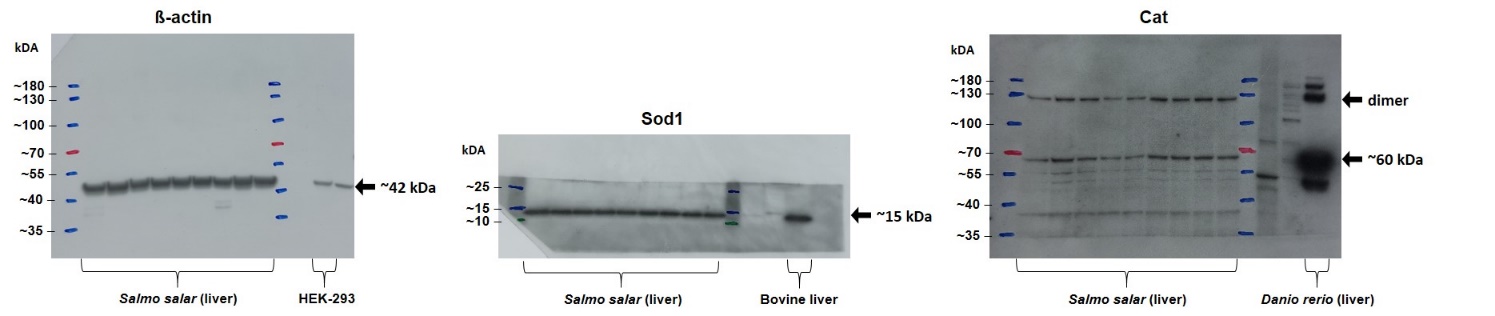


Figure S1. Western Blots with the indication of the protein bands for ß-actin with a predicted mass of ~42 kDA in Atlantic salmon (UniProt: O42161, 41,78 kDa, *S. salar* ß-actin), for Sod1 with a predicted mass of ~15 kDA (UniProt: Q3ZLR1, 15,83 kDa*, S. salar* superoxide dismutase [Cu-Zn]) and Cat with a predicted mass of ~60 kDa per subunit (UniProt: A0A1S3KLQ6*,* 59,48 kDa per subunit, *S. salar* catalase). The blots show samples from salmon liver as well as the respective positive control per antibody (*Danio rerio* liver for Cat, Bovine liver for Sod1 and human HEK-293 cells for ß-actin).


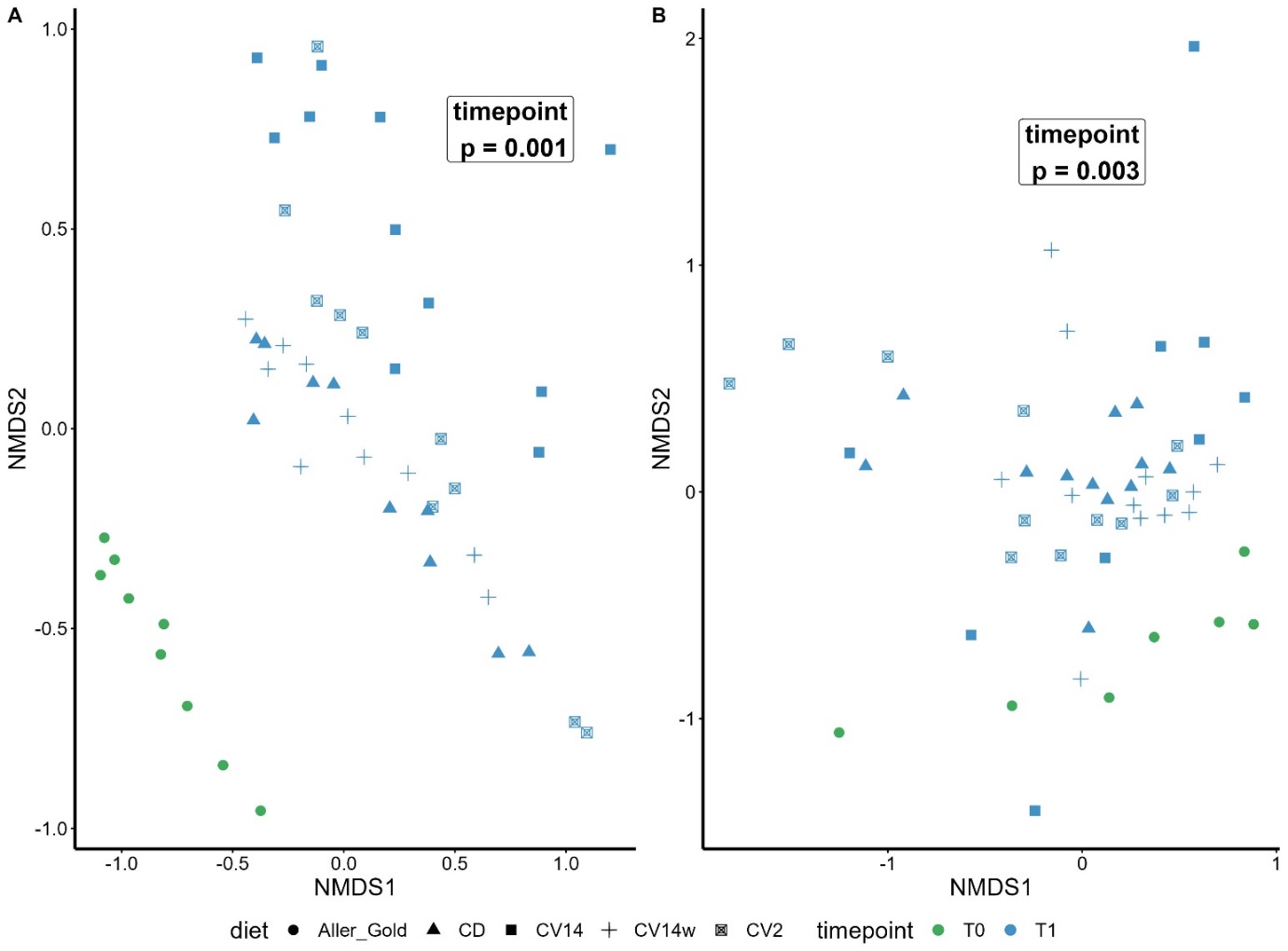


**Figure S2**. Non-metric multidimensional scaling on unweighted UniFrac distances based on weighed genus-level data revealed significant differences between timepoints for both digesta (**A**) und intestinal mucosa (**B**) samples according to PERMANOVA.


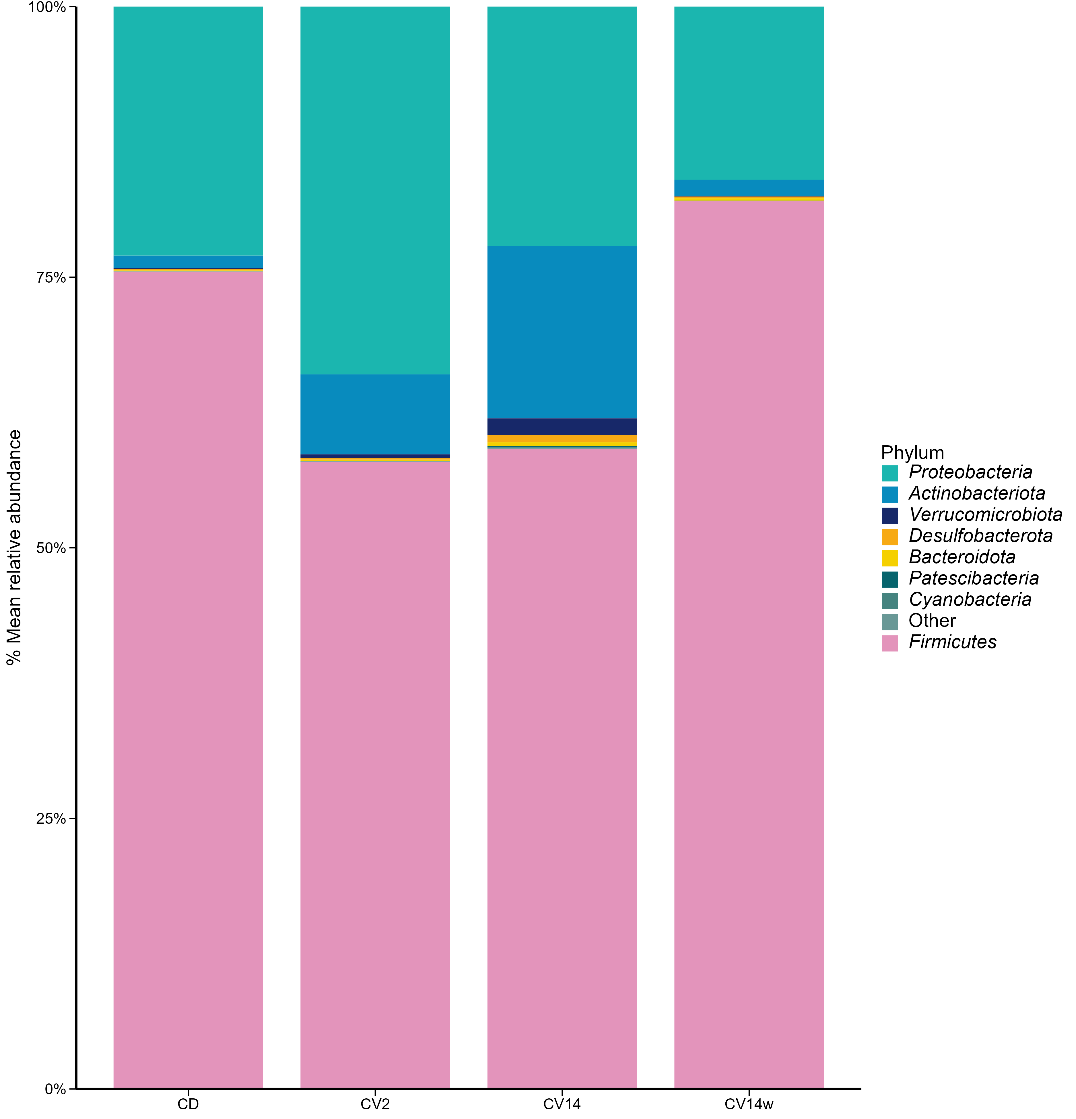


Figure S3. Relative abundance of phyla in the digesta of Atlantic salmon fed diets enriched with *C. vulgaris*. Salmon received either a control diet (CD), a diet with 2% (CV2) or 14% *C. vulgaris* (CV14) on a daily basis, or a diet containing 14% *C. vulgaris* once weekly for eight weeks.


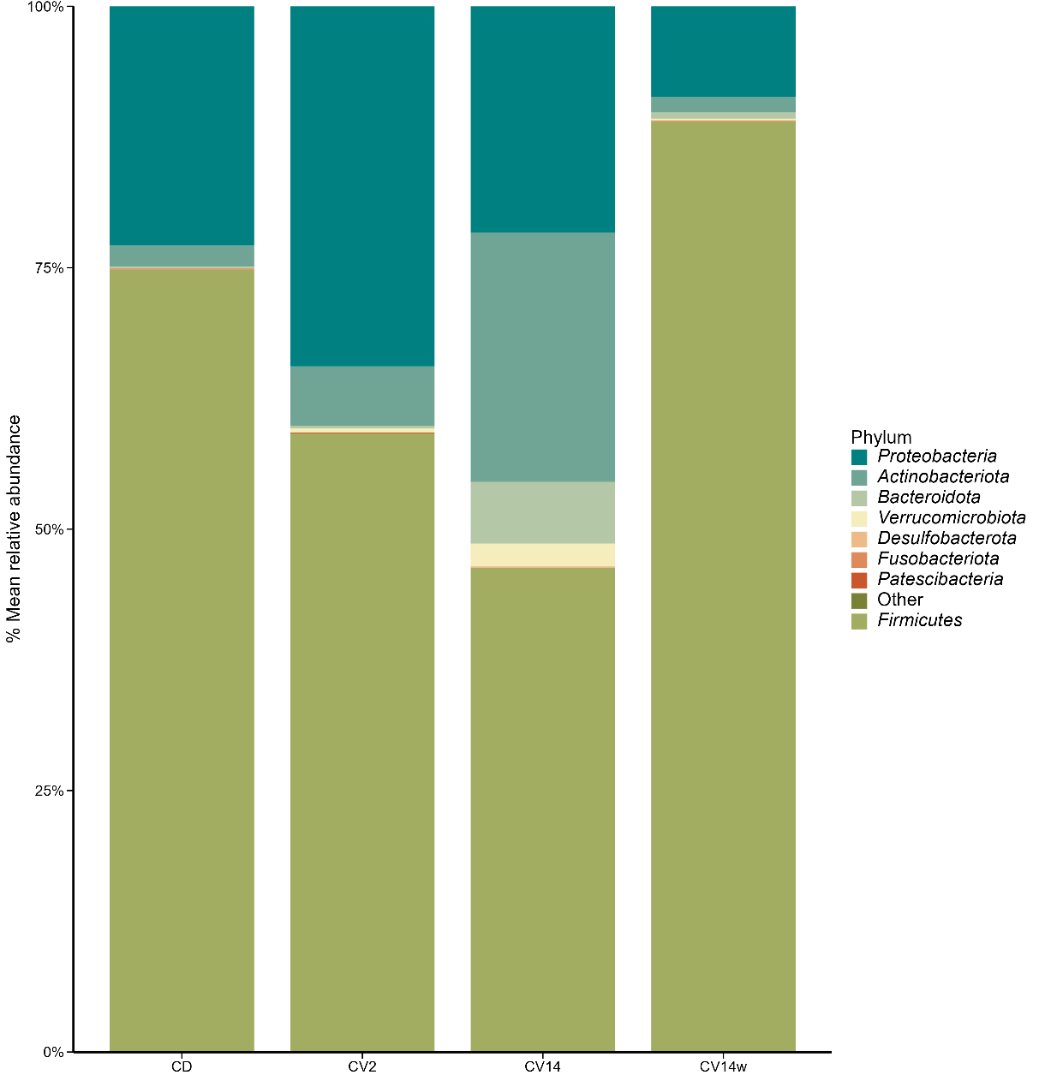


Figure S4. Relative abundance of phyla in the intestinal mucosa of Atlantic salmon fed diets enriched with *C. vulgaris*. Salmon received either a control diet (CD), a diet with 2% (CV2) or 14% *C. vulgaris* (CV14) on a daily basis, or a diet containing 14% *C. vulgaris* once weekly for eight weeks.

**B**

**A**


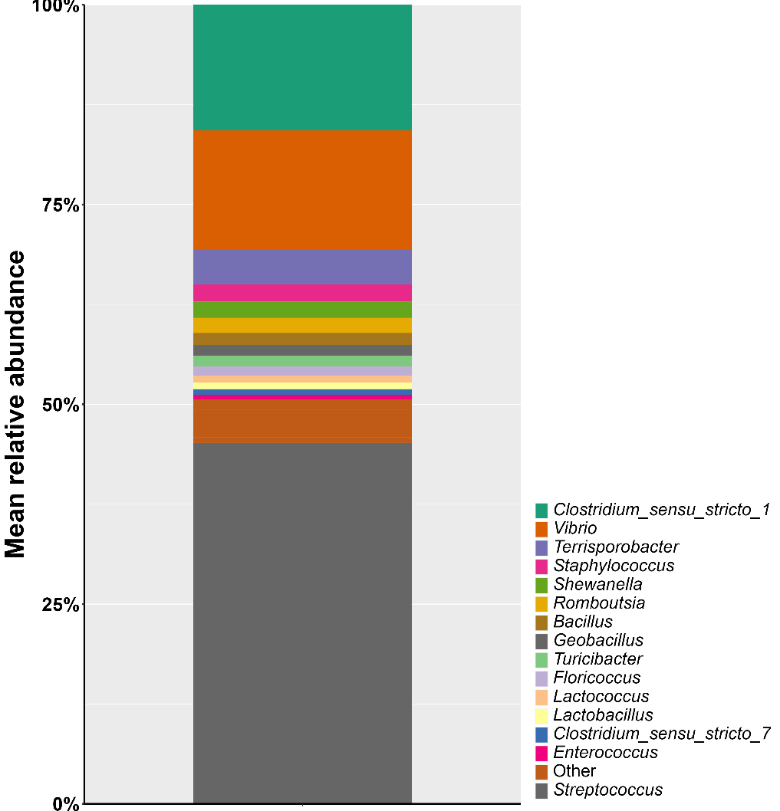

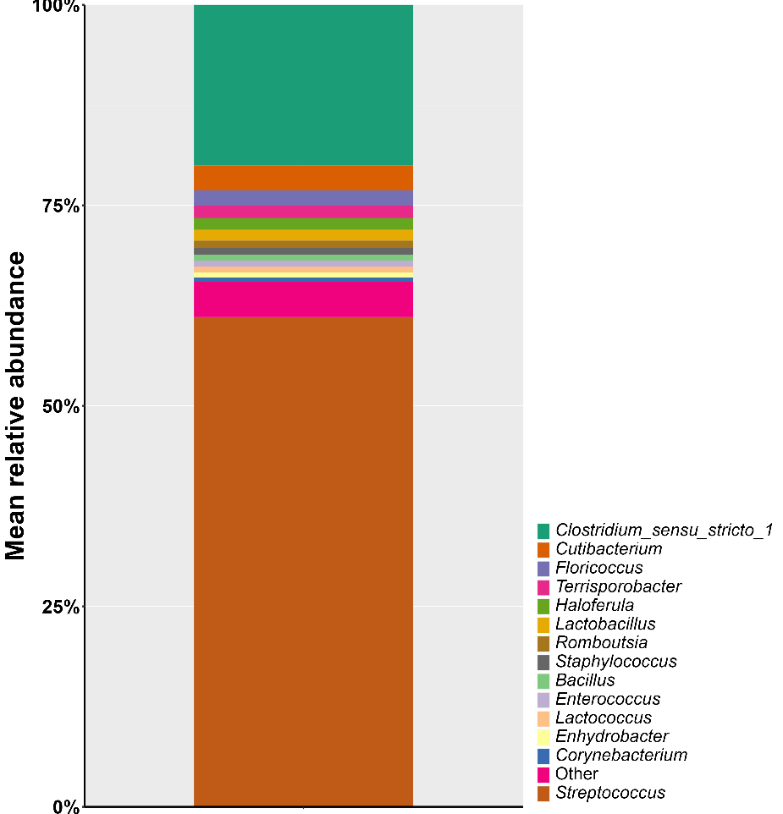


Figure S5. Initial microbial composition at the genus level of the intestinal mucosa (A) and digesta (B) of Atlantic salmon fed a commercial diet; n = 9.


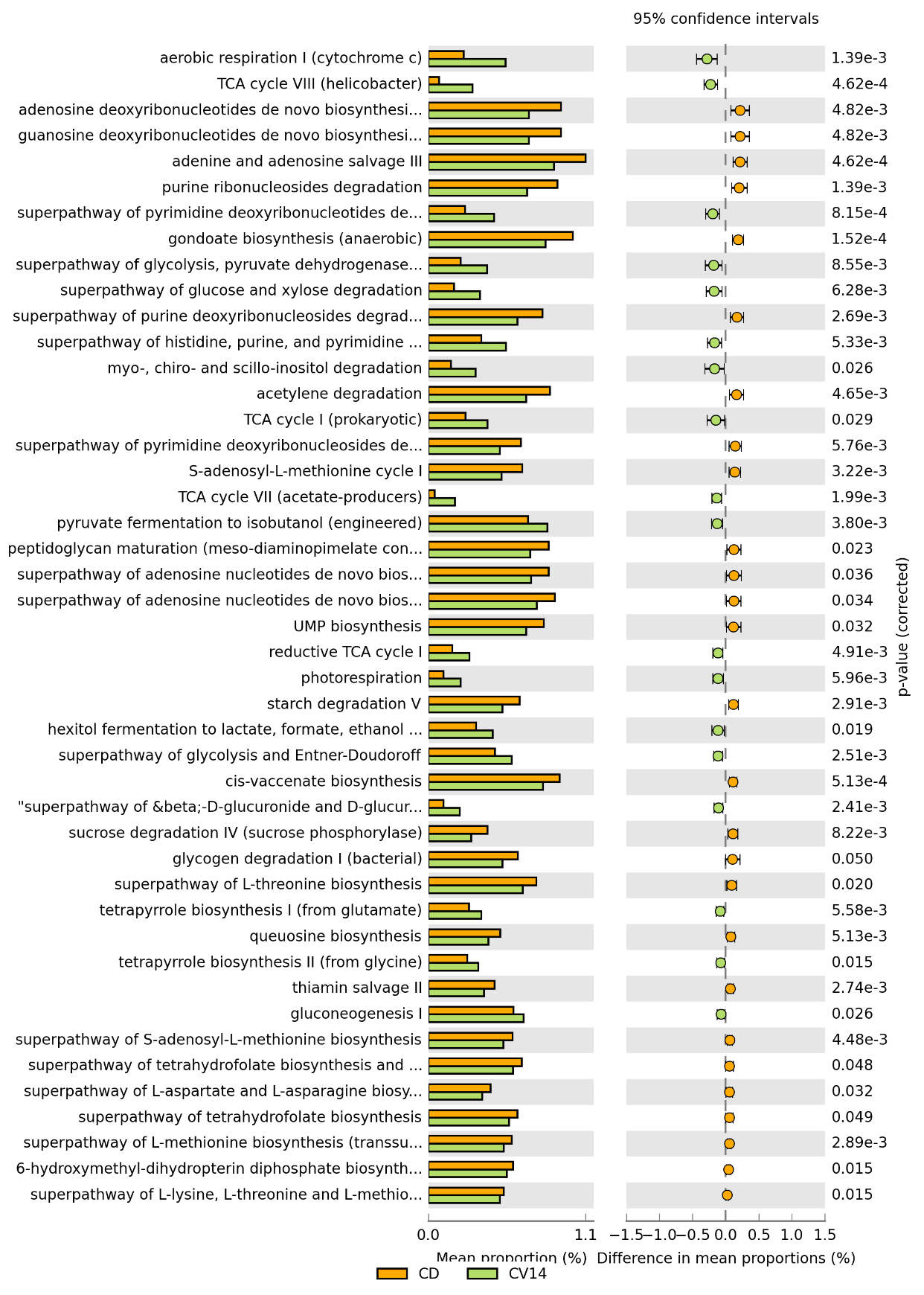


Figure S6. Significant metabolic pathways in the digesta between fish fed CV14 and CD based on PICRUSt2 analysis. Metabolic pathway information was inferred from the metacyc database and sorted after effect size.


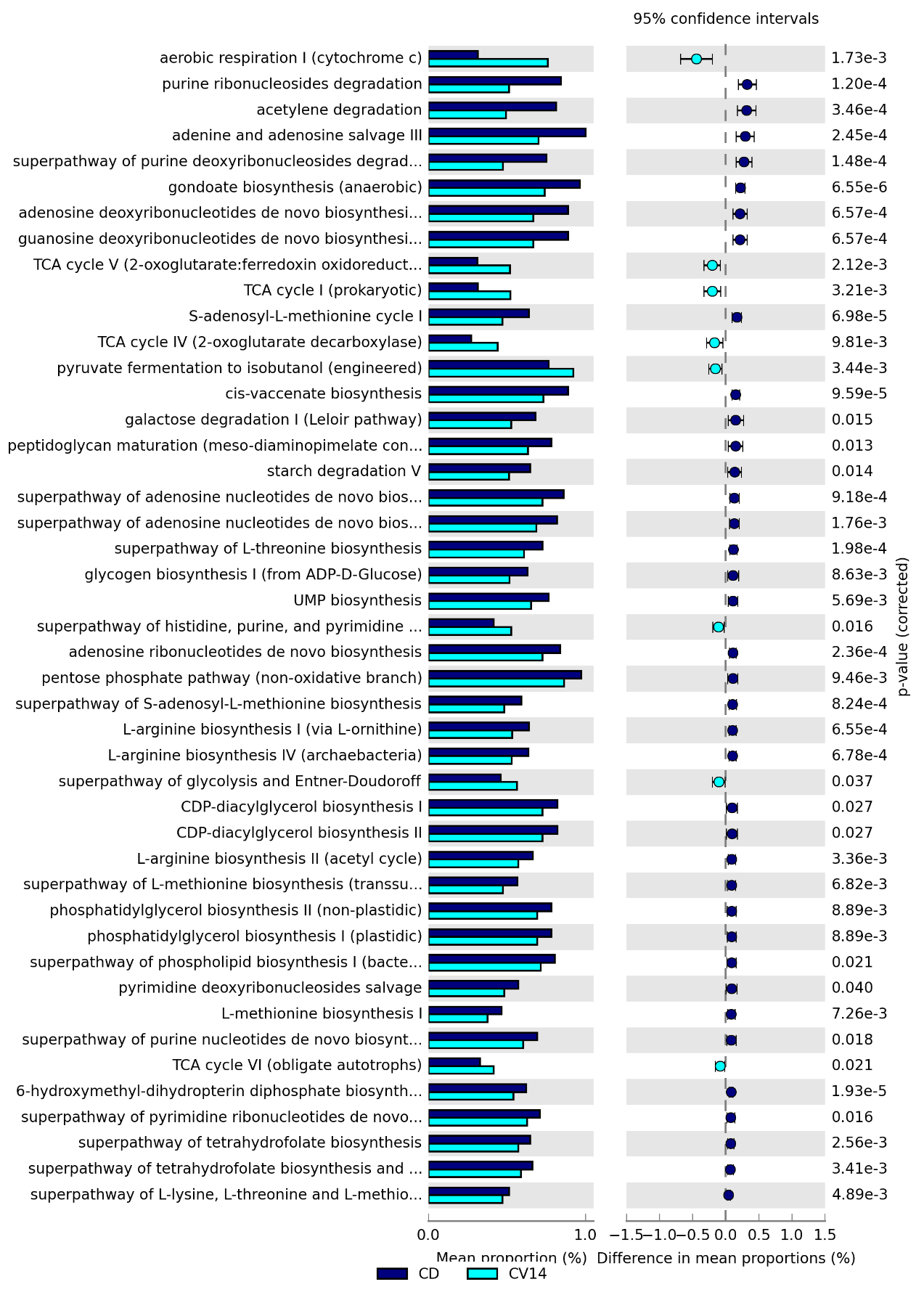


Figure S7. Significant metabolic pathways in the digesta between fish fed CV14 and CD based on PICRUSt2 analysis. Metabolic pathway information was inferred from the metacyc database and sorted after effect size.
